# Diastereoselective Synthesis of *cis*-2,6-Disubstituted Dihydropyrane Derivatives through a Competitive Silyl-Prins Cyclization versus Alternative Reaction Pathways

**DOI:** 10.3390/molecules28073080

**Published:** 2023-03-30

**Authors:** Laura F. Peña, Enol López, Ángel Sánchez-González, Asunción Barbero

**Affiliations:** 1 Department of Organic Chemistry, Campus Miguel Delibes, University of Valladolid, 47011 Valladolid, Spain; 2 BioISI-Biosystems and Integrative Sciences Institute, Departamento de Química e Bioquímica, Faculdade de Ciências, Universidade de Lisboa, Campo Grande, 1749-016 Lisboa, Portugal

**Keywords:** Prins cyclization, heterocycles, oxacycles

## Abstract

A convenient regioselective synthesis of allyl- and vinylsilyl alcohols, from a common precursor, was described, by selecting the appropriate reaction conditions. Allyl- and vinylsilyl alcohols were tested in silyl-Prins cyclizations for the preparation of disubstituted oxygenated heterocycles in a one-pot sequential reaction. The methodology was sensitive to the structure of the starting alkenylsilyl alcohol and reaction conditions, with competitive pathways observed (particularly for allylsilyl alcohols), such as Peterson elimination and oxonia-Cope reactions. However, the use of vinylsilyl alcohols allowed the preparation of differently disubstituted *cis*-2,6-dihydropyrans in moderate to good yields. Computational studies support the proposed mechanism.

## 1. Introduction

Heterocyclic compounds are some of the most common chemical structures found in nature. Among them, functionalized oxygen heterocycles have attracted the attention of scientists, due to their presence in a wide number of biologically active natural products [1,2]. Tremendous efforts have been made to develop synthetic methodologies which can contribute to the construction of these cores. Particularly, intramolecular Prins cyclization has been established as a powerful tool in organic synthesis, for the construction of five- to eight-membered oxacycles, serving as key intermediates for the total synthesis of a variety of natural products [3]. This methodology involves the reaction of an alkenol with an aldehyde, in the presence of a Brønsted or Lewis acid. The general accepted mechanism involves the generation of an initial oxocarbenium ion intermediate, which can subsequently evolve through an *endo*-*dig* cyclization. Then, the corresponding cyclic carbocation can be trapped with a nucleophile, present in the reaction medium. High stereoselectivities are usually obtained, due to the equatorial disposition of substituents in chairlike transition states (TS).

**Scheme 1:**
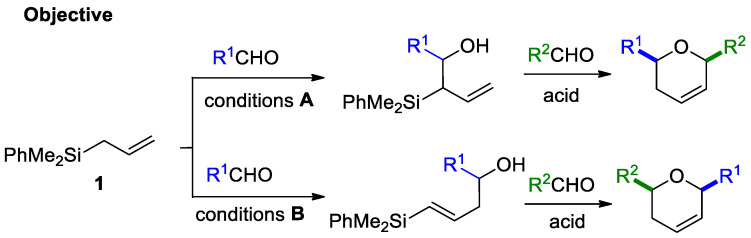
Regioselective formation of allyl- and vinyl alcohols and subsequent Prins cyclization.

Due to the importance of these types of oxacycles in drug discovery, new chemical modifications have been studied in recent decades, to refine and improve their synthesis. The so-called silyl-Prins cyclization, involves the participation of electron-rich alkenes, such as allyl- or vinylsilanes, and shows better selectivity towards the formation of single products, due to the stabilization of the carbocation β to silicon [4,5,6]. Although this methodology has been reported for some allylsilyl [7,8,9,10] and vinylsinyl alcohols [11,12,13,14,15], more efforts are required to increase the reaction scope and facilitate the access to novel 2,6-disubstituted dihydropyrane derivatives. Based on our experience in silyl-Prins cyclizations [16,17,18], in this work we investigate the access to either allyl- or vinylsilyl homoallylic alcohols from a common allyl(dimethyl)phenylsilane, and the possibilities of participation of the resulting substrates in silyl-Prins cyclizations (Scheme 1). Considering previously reported methodologies, as well as our model substrate, we anticipate some challenges that must be overcome:

(a) Selective methodologies are needed to obtain exclusively allyl- or vinyl silylalcohols from a common allylsilane precursor (conditions **A** and **B**, Scheme 1).
(b) Alternative reaction pathways may interfere in the silyl-Prins cyclization reaction outcome [19], generating complex mixtures due to the formation of highly reactive intermediates.

Our first goal, is the regioselective synthesis of new allyl- and *E*-vinylsilyl homoallylic alcohols, from a common allylsilane **1**. Once these reaction conditions have been optimized, attempts to achieve silyl-Prins cyclization from both derivatives are evaluated, in order to compare the selectivity and the scope of both convergent processes. In addition, we study (both by computational and experimental methods) the challenge of favoring silyl-Prins cyclization over alternative reaction pathways.

## 2. Results

### 2.1. Synthesis of Allyl- and Vinylsilyl Alcohols

We started our investigations synthesizing allyl(diphenyl)silane **1**. This substrate was prepared with the in situ formation of an organozinc reagent from allyl bromide, which was subsequently added to a solution of chloro(dimethyl)phenylsilane in THF at 0 °C (Section 3.2.2) [20]. The desired derivative **1** was obtained in a 95% yield and could be used without any further purification. With **1** in hand, we focused our attention on the regioselective synthesis of allyl- and vinylsilyl alcohols **2** and **3**. The acidic character of the protons α to the silicon atom, could be used to generate an α-silylcarbanion, that was in equilibrium with a γ-silylcarbanion. Then, subsequent addition of an aldehyde under controlled conditions exclusively generated the desired derivatives (**2** or **3**).

**Scheme 2:**
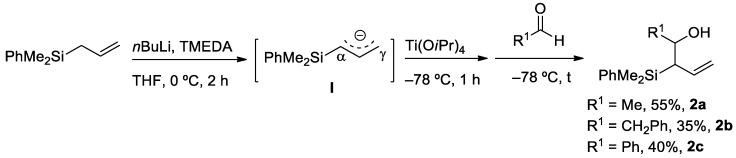
Regioselective synthesis of allylsilyl alcohols **2**.

Allylsilyl alcohols **2** were obtained when the incorporation of the electrophile occured though the α-position. Thus, the reaction of **1** with *n*-BuLi, in the presence of *N*-tetramethylethylendiamine (TMEDA), generated an intermediate silylcarbanion **I**. Ti(O*^i^*Pr)_4_ was subsequently added as an auxiliary reagent, to block the γ-position and promote the selective addition through the α-position (Scheme 2). In this manner, various homoallylic alcohols **2** were selectively prepared in moderate yields, by addition of either alkyl- or arylaldehyde coupling partners (**2a–c**, 35–55%). The reaction takes places with total diastereoselectivity, obtaining exclusively *anti* products [21].

**Scheme 3:**
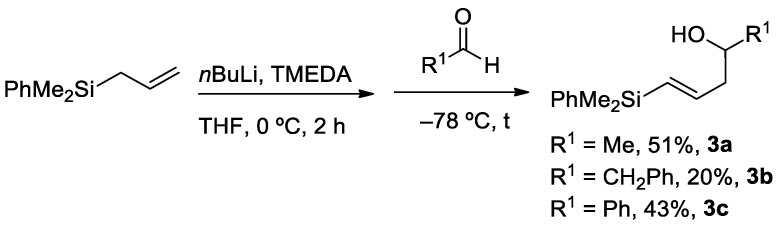
Regioselective synthesis of vinylsilyl alcohols **3**.

Fortunately, in the absence of the auxiliary reagent Ti(O*^i^*Pr)_4_, the intermediate silylcarbanion **I**, reacts with aldehydes through the desired γ-position, which is the least hindered one, providing the regioisomeric vinylsilyl alcohols **3**. Representative examples were obtained with both alkyl- and aryl aldehydes, in moderate yields (Scheme 3). With these alternative protocols we were able to prepare a series of compounds **2** and **3**, ready to be used in silyl-Prins cyclization reactions.

### 2.2. Study of the Silyl-Prins Cyclization with Allylsilyl Alcohols **2**

With various allylsilyl alcohols **2** in hand, we screened a series of reaction conditions, to achieve the desired silyl-Prins cyclization.

**Scheme 4:**
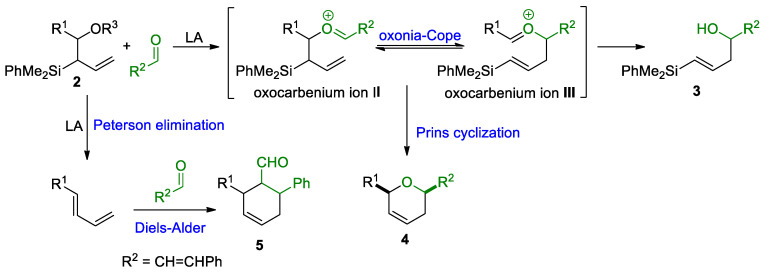
Reaction pathways from allylsilyl alcohols **2**.

We started our investigations with derivative **2a** (R^1^ = Me), which was treated with various Lewis acids (1.2 equiv.) at different reaction temperatures (Table 1, entries 1–4). Unfortunately, we did not observe the formation of the desired oxacycle **4**, obtaining complex mixtures, and identifying product **3** at low temperature (Table 1, entry 1). The formation of vinylsilyl alcohol **3**, would imply a competitive oxonia-Cope reaction of intermediate **II**, instead of the desired cyclization step (Scheme 4). To minimize side-reactions, we decided to use less reactive aldehydes, such as (*E*)-3-phenylprop-2-enal (Table 1, entry 5). However, ^1^H-NMR analysis of the reaction crude, showed a complex mixture, in which unreacted aldehyde was observed, but neither the starting material nor oxonia-Cope products could be identified. This result suggests the possibility of an alternative reaction pathway from **2**, such as a Peterson elimination, which would provide a volatile 1,3-pentadiene, probably lost in the rotavapor system (Scheme 4). To further study the occurrence of this competitive reaction, we set up the reaction with allylsilyl alcohols **2b** (R^1^ = CH_2_Ph) and **2c** (R^1^ = Ph) (Table 1, entries 6–9). Gratifyingly, a detailed analysis of the reaction mixture now showed the presence of cyclic products (**4a** or **4i**), together with **3a** (Table 1, entries 6 and 7), which suggested an initial oxonia-Cope reaction, followed by subsequent cyclization. In order to try to overcome secondary reactions, we turned our attention to the use of trimethylsilyl ester derivatives as starting materials (Table 1, entries 10–12) [22]. Unfortunately, no reaction, or complex mixtures, was observed (Table 1, entries 10 and 11), although an interesting new product **5** was identified and characterized (Table 1, entry 12). The formation of this carbocycle can be explained through a Diels–Alder reaction of the 1,3-diene produced in the Peterson elimination and the corresponding aldehyde ((*E*)-3-phenylprop-2-enal), which corroborates the previously proposed Peterson elimination pathway (Scheme 4).

As shown before, the reaction of different allylsilyl alcohols with aldehydes, in the presence of Lewis acids, turned out to be very challenging, due to the different behavior of the implicated substrates, as well as the variety of competitive reaction pathways. However, we were able to identify and isolate key products for a better understanding of the implicated intermediates, that will help in the development of future silyl-Prins cyclizations. Next, and considering previous data, we focused our attention on the study of the silyl-Prins cyclization of vinylsilyl alcohols **3**.

### 2.3. Silyl-Prins Cyclization Study of Vinylsilyl Alcohols **3**

First of all, we screened different conditions and Lewis acids, in order to find the optimized conditions for the cyclization, using as a key model derivative **3a**. As shown in Table 2, complex mixtures were obtained at 0 °C when BF_3_·OEt_2_ or TMSCl were used as Lewis acids (Table 2, entries 1–4). Gratifyingly, a *cis*-2,6-disubstituted dihydropyrane derivative **4a** was obtained when 1 equiv. of TMSOTf was used at −78 °C (Table 2, entries 5–7). Analysis of the reaction crude, showed very good diastereoselectivity for *cis*-**4a** (*cis:trans*, 90:10), which was isolated in a 48% yield (Table 2, entry 6). Increasing the equivalents of the aldehyde, or reducing the amount of the Lewis acid, provided lower yields (Table 2, entries 5 and 7).

**Scheme 5:**
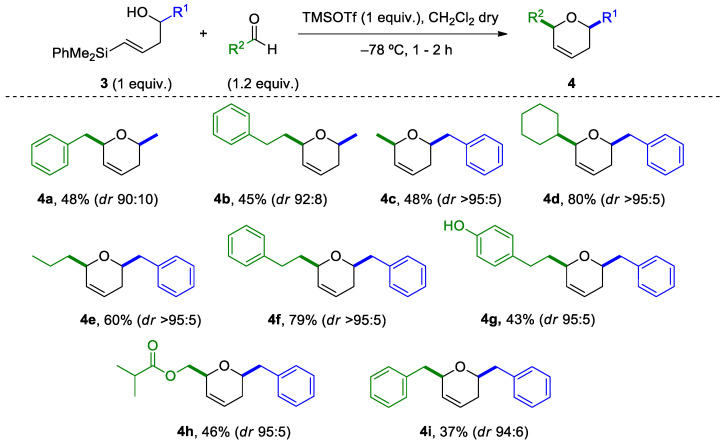
Reaction scope of the silyl-Prins cyclization.

Having determined the optimal conditions, we studied the reaction scope for other vinylsilyl alcohols and aldehydes (Scheme 5). As shown, the reaction of (E)-vinylsilyl alcohols bearing an alkylic R^1^ group, with alkylic aldehydes, provides, in moderate to good yields, the desired disubstituted dihydropyrans (**4a–4g**). Interestingly, although the reaction of allylsilyl alcohols **2** with aldehydes failed to give the desired oxacycles (Table 2), we now were able to circumvent this difficulty by choosing the appropriate substituents in the vinylsilyl alcohols and the aldehyde (for instance, dihydropyran **4b** could be obtained using the homologated aldehyde partner). It was also possible to install a phenylethylene group in the dihydropyrane with good diastereoselectivity (*dr* > 95:5), with the preparation of compound **4f** (79% yield). However, the presence of a hydroxy group in the aromatic ring of the aldehyde (when the corresponding 3-(*p*-hydroxyphenyl)propionaldehyde was used) significantly affected the yield of the reaction (**4g**, 43%). Other interesting functional groups, such as an isopropylester, could also be installed successfully (**4h**, 46% yield). An interesting aspect of this reaction, is the excellent diastereoselectivity observed in most cases for the formation of the *cis*-2,6-disubstituted dihydropyran. On the other hand, less reactive aromatic or vinylic aldehydes produced complex mixtures, in which it was possible to isolate symmetric tetrahydropyrans, with identical substituents attached at the 2,6-positions (**4i**, 37% yield). This is in accordance with our previous results on the reaction with vinylsilyl alcohols, in which an alternative sigmatropic [3,3] oxonia-Cope transposition pathway was more favored. Similarly, vinylsilyl alcohols with an aryl R^1^ group, provided complex reaction mixtures.

### 2.4. Reaction Mechanism Study

**Scheme 6:**
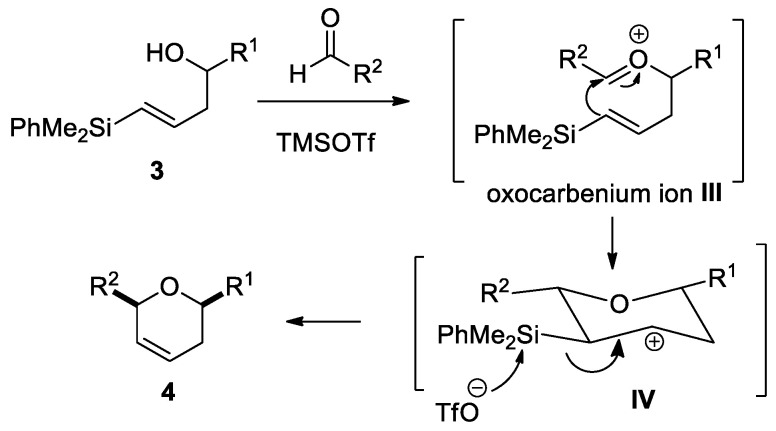
Mechanism proposal of the silyl-Prins cyclization.

The reaction of allylsilyl alcohols turned out to be more challenging than the corresponding one with *E*-vinylsilyl alcohols. Very likely, this is due to the concurrence of more favored alternative pathways, such as oxonia-Cope transposition and Peterson elimination. In the case of *E*-vinylsilyl alcohols, *cis*-dihydropyrane derivatives were selectively obtained through a silyl-Prins cyclization reaction. A plausible mechanism for this reaction would start with the reaction of *E*-vinylsilyl alcohol **3** with the aldehyde, in the presence of a Lewis acid, to generate an oxocarbenium ion **III** (Scheme 6). Then, a 6-*endo-dig* cyclization would take place, to afford intermediate **IV** (stabilized by the silicon atom), followed by a subsequent desilylation reaction, to afford final product **4** [23]. This process can be considered a tandem reaction, in which TMSOTf promotes the formation of intermediate **III** and the elimination step. The stereocontrol can be explained through the preferential pseudoequatorial conformation of substituents R^1^ and R^2^ in the transition state, to achieve minimal repulsion.

In order to gain insight into the reaction mechanism and the high stereoselectivity observed, a computational study of the silyl-Prins cyclization has been performed at a fundamental level. In this work, the Amsterdam Density Functional 2017.01 (ADF) [24,25] software package was used to perform DFT calculations. The geometries were fully optimized without symmetry constraints, using the combination of the gradient corrections of the Becke exchange functional and Perdew correlation functional, which use the Vosko–Wilk–Nusair exchange-correlation potential reported by Becke (1998) and Perdew (1986) (BP86-D3) [26,27,28], in combination with the latest version of Grimme dispersion correction [29]. Triple-ζ Slater-type orbitals (STO), were used to describe the valence shells. The solvation environment was treated with the Conductor-like Screening Model (COSMO) [30], considering dichloromethane (ɛ = 8.9) in order to reproduce the experimental conditions performed in our lab. Analytical frequencies were computed, to characterize the stationary points and calculate the free energies (standard state T = 298.15 K, *p* = 1 atm). The transition state was followed after a fractional displacement of the imaginary vibrational mode, to determine the reaction path to reactant(s) and product(s). Molecular renderings were made with Chemcraft [31].

**Figure 1:**
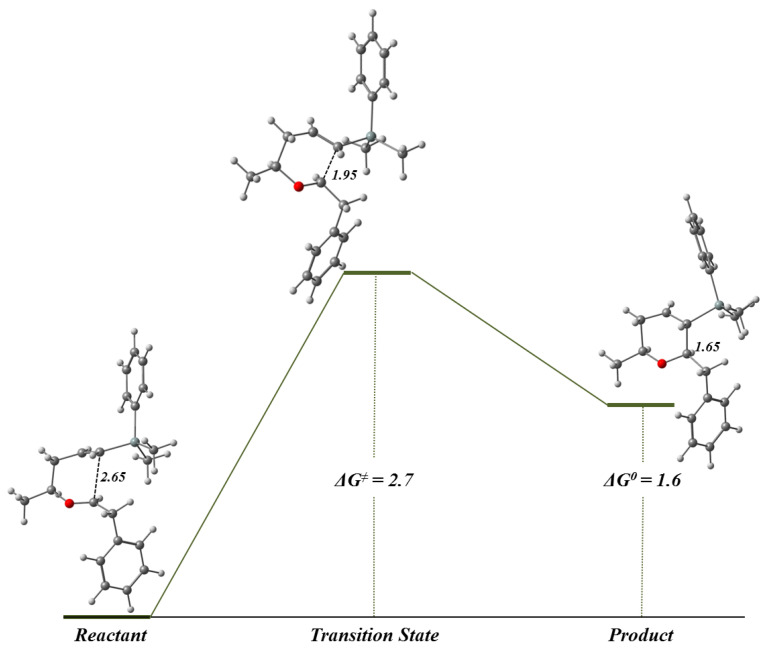
Reaction scheme for silyl-Prins cyclization. Gibbs free energies are provided in kcal·mol^−1^, and distances in angstroms (Å).

In Figure 1, the reaction profile of the 6-*endo* silyl-Prins cyclization towards dihydropyran **4a** is shown. In this study, only the approximation between the two carbon atoms that yields a chair conformation for the oxygenated heterocycle is described, since no transition state has been found for a boat conformation. The results obtained, indicate that the reaction shows a very low energy profile, with an energy barrier for the transition state of 2.7 kcal/mol. In such a transition state, the distance between the C atoms which will be bonded in the cyclization step, corresponds to 1.95 Å. Following the imaginary vibrational mode to cyclic intermediate **IV** (tetrahydropyranyl carbocation), the minimum presents a Gibbs free energy value of 1.6 kcal/mol, which corresponds to an energy difference with the transition state of only 1.1 kcal/mol. This low energy profile is consistent with the experimental results concerning the reversibility of the silyl-Prins cyclization, which could follow other competitive pathways. Furthermore, we carried out computational studies for the *R* isomer (C atom that holds the methyl group); geometries of the reactants and products were fully optimized. The obtained results, show that for the *R* isomer, where the methyl is in axial arrangement, both ΔH and ΔG energies present higher values in comparison with the *S* isomer (see Supporting Information, Figure S1). In addition, for the *R* isomer, the rotational freedom allowed by the sigma bonds prior to cyclization could lead to another conformation, where the bulky groups (methyl and benzyl) are placed in equatorial arrangement, which explains the preferential formation of *cis*-2,6-disubstituted derivatives **4** (see Supporting Information, Scheme S1).

## 3. Materials and Methods

### 3.1. General Remarks

Unless otherwise noted, experiments were carried out with dry solvents under a nitrogen atmosphere. Dichloromethane was dried with preactivated molecular sieves. Flash column chromatography was performed using Silica Gel 60 (230–400 mesh ASTM). Thin-layer chromatography (TLC) was performed using an aluminum backed plate, pre-coated with silica gel (0.20 mm, silica gel 60), with a fluorescent indicator (254 nm) from Macherey. NMR spectra were recorded at the nuclear magnetic resonance service of the Laboratory of Instrumental Techniques (L.T.I., www.laboratoriotecnicasinstrumentales.es), University of Valladolid, at Varian 400 MHz (^1^H, 399.85 MHz; ^13^C, 100.61 MHz) and Varian 500 MHz (^1^H, 500.12 MHz; ^13^C, 100.61 MHz), with the spectrometers at room temperature (25 °C). Chemical shifts (δ) were reported in parts per million (ppm), relative to the residual solvent peaks recorded, rounded to the nearest 0.01 for ^1^H-NMR and 0.1 for ^13^C-NMR (reference: CDCl_3_ [^1^H: 7.26, ^13^C: 77.2]). Spin–spin coupling constants (*J*) in ^1^H-NMR were given in Hz, to the nearest 0.1 Hz, and peak multiplicity was indicated as follows s (singlet), d (doublet), t (triplet), q (quartet), m (multiplet), and br (broad). ^13^C-NMR were recorded with complete proton decoupling. Carbon types, structure assignments and attribution of peaks were determined from two-dimensional correlation experiments (HSQC, COSY, and HMBC). Relative stereochemistry was assigned based on the 2D-NOE experiments. High-resolution mass spectra (HRMS) were measured at the mass spectrometry service of the Laboratory of Instrumental Techniques, University of Valladolid, on a UPLC-MS system (UPLC: Waters ACQUITY H-class UPLC; MS: Bruker Maxis Impact) by electrospray ionization (ESI positive and negative).

### 3.2. Synthesis of Allyl(diphenyl)silane **1**

A solution of 2.99 g (45 mmol, 1.5 equiv.) of zinc, in 25 mL THF (1.2 M), was cooled to 0 °C under a nitrogen atmosphere. Then, allyl bromide (45 mmol, 1.5 equiv.) was added, and after five minutes under vigorous stirring, 5.03 mL of phenyldimethylchlorosilane (30 mmol, 1.0 equiv.) was added dropwise. When the starting materials had been consumed, it was hydrolyzed with 20 mL of NH_4_Cl sat. The phases were then separated, extracting the aqueous phase three times with diethyl ether. The organic phases were combined, washed with NaCl sat., and dried over anhydrous Na_2_SO_4_. The solvent was then evaporated under reduced pressure. The crude mixture was analyzed by NMR and then purified by column chromatography in silica gel, using hexane, providing allylsilane **1** in quantitative yield. This (**1**) was obtained as a colorless oil in 95% chemical yield (5.02 g from 30 mmol of PhMe_2_SiCl). ^1^H-NMR (500 MHz, CDCl_3_) δ 7.52–7.47 (m, 2H, Ar-*H*), 7.36–7.32 (m, 3H, Ar-*H*), 5.83–5.71 (m, 1H, *H*C=), 4.87 (d, *J^trans^* = 17.0 Hz, 1H, =C*H*H), 4.83 (d, *J^cis^* = 10.4 Hz, 1H, = CH*H*), 1.74 (d, *J* = 8.0 Hz, 2H, C*H_2_*), 0.27 (s, 6H, (C*H_3_*)_2_- Si).

### 3.3. Synthesis of Allylsilyl Alcohols **2**

A solution of 1.5 g (8.5 mmol, 1.0 eq.) of allylsilane **1**, in 15 mL THF (0.57 M), was cooled to 0 °C (under nitrogen). Next, 6.4 mL n-BuLi (10.2 mmol, 1.1 eq.) and 2.1 mL TMEDA (14.0 mmol, 1.65 eq.) were added dropwise. The mixture was stirred at this temperature for 2 h. Then, the reaction as cooled down to −78 °C and 2.52 mL Ti(^i^OPr)_4_ (8.5 mmol, 1.0 eq.) was added. After 1 h, the corresponding aldehyde (10.2 mmol, 1.2 eq.) was added dropwise to the solution. When the starting materials had been consumed (control by TCL), it was hydrolyzed with 15 mL of NH_4_Cl sat. The phases were then separated, extracting the aqueous phase three times with diethyl ether. The organic phases were combined, washed with NaCl sat., and dried over anhydrous Na_2_SO_4_. The solvent was then evaporated under reduced pressure. The crude mixture was analyzed by NMR and then purified by column chromatography in silica gel, using a mixtures of hexane-ethyl acetate (10:1), yielding allylsilyl alcohols **2**.

*3-(dimethyl(phenyl)silyl)pent-4-en-2-ol* (**2a**) was obtained following the general procedure 3.3, as a yellow oil, in 55% chemical yield (3.028 g from 22.7 mmol of **1**). ^1^H-NMR (500 MHz, CDCl_3_) δ 7.56–7.49 (m, 2H, Ar-*H*), 7.39–7.32 (m, 3H, Ar-*H*), 5.81 (dt, *J* = 17.2, 10.4 Hz, 1H, *H*C=), 5.09 (dd, *J* = 10.4, 1.7 Hz, 1H, =C*H*H), 4.96 (ddd, *J* = 17.2, 2.0, 0.7 Hz, 1H, =CH*H*), 4.00–3.87 (m, 1H, *H*C-OH), 1.85 (dd, *J* = 10.4, 6.0 Hz, 1H, *H*C), 1.52 (br s, 1H, O*H*), 1.11 (d, *J* = 6.3 Hz, 3H, C*H_3_*), 0.35 (s, 3H, Si-C*H_3_*), 0.32 (s, 3H, Si-C*H_3_*). ^13^C-NMR (101 MHz, CDCl_3_) δ 138.0 (C), 135.9 (HC=), 134.1 (CH), 129.2 (CH), 127.9 (CH), 116.1 (=CH_2_), 67.6 (HC-OH), 44.7 (CH), 23.6 (CH_3_), −3.0 (Si-CH_3_), −3.8 (Si-CH_3_).

*3-(dimethyl(phenyl)silyl)-1-phenylpent-4-en-2-ol* (**2b**) was obtained following the general procedure 3.3, as a yellow oil, in 33% chemical yield (826 mg from 8.5 mmol of **1**). ^1^H-NMR (500 MHz, CDCl_3_) δ 7.56–7.49 (m, 2H, Ar-*H*), 7.37–7.31 (m, 3H, Ar-*H*), 7.29–7.24 (m, 3H, Ar-*H*), 7.23–7.17 (m, 1H, Ar-*H*), 7.12–7.08 (m, 2H, Ar-*H*), 5.97 (dt, *J* = 17.1, 10.5 Hz, 1H, *H*C=), 5.11 (dd, *J* = 10.4, 2.1 Hz, 1H =C*H*H), 4.92 (ddd, *J* = 17.1, 2.1, 0.5 Hz, 1H, =CH*H*), 4.01–3.90 (m, 1H, *H*C-OH), 2.70–2.65 (m, 2H, Ph-C*H_2_*), 1.96 (dd, *J* = 10.4, 3.5 Hz, 1H, C*H*), 1.45 (dd, *J* = 3.5, 1.1 Hz, 1H, O*H*), 0.36 (s, 3H, Si-C*H_3_*), 0.32 (s, 3H, Si-C*H_3_*). ^13^C-NMR (101 MHz, CDCl_3_) δ 139.1 (C), 138.0 (C), 135.1 (HC=), 134.2 (CH), 129.4 (CH), 129.2 (CH), 128.6 (CH), 127.9 (CH), 126.4 (CH), 115.7 (=CH_2_), 72.6 (HC-OH), 44.0 (CH_2_), 41.6 (CH), −3.4 (Si-CH_3_), −3.8 (Si-CH_3_).

*2-(dimethyl(phenyl)silyl)-1-phenylbut-3-en-1-ol* (**2c**) was obtained following the general procedure 3.3, as a yellow oil, in 49% chemical yield (1.18 g from 8.5 mmol of **1**). ^1^H-NMR (500 MHz, CDCl_3_) δ 7.48–7.42 (m, 2H, Ar-*H*), 7.37–7.31 (m, 3H, Ar-*H*), 7.29–7.25 (m, 2H, Ar-*H*), 7.24–7.20 (m, 3H, Ar-*H*), 5.88 (dt, *J* = 17.2, 10.4 Hz, 1H, *H*C=), 5.05 (dd, *J* = 10.4, 1.8 Hz, 1H, =C*H*H), 4.90 (dd, *J* = 17.2, 1.8 Hz, 1H, =CH*H*), 4.75 (dd, *J* = 6.9, 2.7 Hz, 1H, *H*C-OH), 2.26 (dd, *J* = 10.4, 6.9 Hz, 1H, *H*C), 2.03 (d, *J* = 2.7 Hz, 1H, O*H*), 0.19 (s, 3H, Si-C*H_3_*), 0.08 (s, 3H, Si-C*H_3_*). ^13^C-NMR (101 MHz, CDCl_3_) δ 143.8 (C), 137.5 (C), 135.8 (HC=), 134.3 (CH), 129.2 (CH), 128.3 (CH), 127.8 (CH), 127.7 (CH), 126.8 (CH), 116.4 (=CH_2_), 74.3 (HC-OH), 45.1 (CH), −3.3 (Si-CH_3_), −3.9 (Si-CH_3_).

### 3.4. Synthesis of Vinylsilyl Alcohols **3**

A solution of 1.5 g (8.5 mmol, 1.0 equiv.) of allylsilane **1** in 15 mL THF (0.57 M) was cooled to 0 °C under a nitrogen atmosphere. Next, 6.4 mL of n-BuLi (10.2 mmol, 1.1 equiv.) and 2.1 mL TMEDA (14.0 mmol, 1.65 equiv.) were added dropwise. The mixture was stirred at this temperature for 2 h. Then, the reaction was cooled down to −78 °C and the corresponding aldehyde (10.2 mmol, 1.2 equiv.) was added dropwise to the solution. When the starting materials had been consumed (control by TLC), it was hydrolyzed with 15 mL of NH_4_Cl sat. The phases were then separated, extracting the aqueous phase three times with diethyl ether. The organic phases were combined, washed with NaCl sat., and dried over anhydrous Na_2_SO_4_. The solvent was then evaporated under reduced pressure. The crude mixture was analyzed by NMR and then purified by column chromatography in silica gel, using a mixtures of hexane-ethyl acetate (8:1), yielding E-vinylsilyl alcohols **3**.

*(E)-5-(dimethyl(phenyl)silyl)pent-4-en-2-ol* (**3a**) was obtained following the general procedure 3.4, as a yellow oil, in 51% chemical yield (1.18 g from 11.4 mmol of **1**). ^1^H-NMR (500 MHz, CDCl_3_) δ 7.53–7.50 (m, 2H, Ar-*H*), 7.37–7.33 (m, 3H, Ar-*H*), 6.11 (dt, *J* = 18.6, 6.7 Hz, 1H, =C*H*), 5.91 (dt, *J* = 18.6, 1.2 Hz, 1H, *H*C=), 3.93–3.84 (m, 1H, *H*C-OH), 2.40–2.33 (m, 1H, C*H*H), 2.32–2.25 (m, 1H, CH*H*), 1.21 (d, *J* = 6.2 Hz, 3H, C*H_3_*), 0.35 (s, 6H, (C*H_3_*)*_2_*-Si). ^13^C-NMR (101 MHz, CDCl_3_) δ 144.8 (=*C*H), 138.9 (C), 133.9 (CH), 132.3 (H*C*=), 129.1 (C-H), 127.9 (CH), 67.0 (H*C*-OH), 46.9 (*C*H_2_), 23.0 (*C*H_3_), −2.4 (*C*H_3_)-Si), −2.5. (*C*H_3_)-Si).

*(E)-5-(dimethyl(phenyl)silyl)-1-phenylpent-4-en-2-ol* (**3b**) was obtained following the general procedure 3.4, as a yellow oil, in 20% chemical yield (500 mg from 8.5 mmol of **1**). ^1^H-NMR (500 MHz, CDCl_3_) δ 7.55–7.50 (m, 2H, Ar-*H*), 7.38–7.35 (m, 3H, Ar-*H*), 7.33–7.29 (m, 2H, Ar-*H*), 7.25–7.20 (m, 3H, Ar-*H*), 6.17 (dt, *J* = 18.7, 6.5 Hz, 1H, =C*H*), 5.94 (d, *J* = 18.7 Hz, 1H, *H*C=), 3.97–3.89 (m,1H, *H*C-OH), 2.82 (dd, *J* = 13.5, 5.1 Hz, 1H, C*H*H-Ph), 2.73 (dd, *J* = 13.5, 7.9 Hz, 1H, CH*H*-Ph), 2.48–2.41 (m, 1H, C*H*H), 2.38–2.29 (m, 1H, CH*H*), 1.66 (br s, 1H, O*H*), 0.35 (s, 6H, Si-(C*H_3_*)_2_). ^13^C-NMR (101 MHz, CDCl_3_) δ 144.7 (=CH), 138.9 (C), 138.5 (C), 133.9 (CH), 132.4 (HC=), 129.6 (CH), 129.1 (CH), 128.7 (CH), 127.9 (CH), 126.6 (CH), 71.7 (HC-OH), 44.4 (CH_2_), 43.5, (CH_2_-Ph), −2.4 (Si-(CH_3_)_2_).

*(E)-4-(dimethyl(phenyl)silyl)-1-phenylbut-3-en-1-ol* (**3c**) was obtained following the general procedure 3.4, as a yellow oil, in 43% chemical yield (890 mg from 7.4 mmol of **1**). ^1^H-NMR (500 MHz, CDCl_3_) δ 7.50–7.44 (m, 3H, Ar-*H*), 7.37–7.32 (m, 7H, Ar-*H*), 6.11 (dt, *J* = 18.6, 6.7 Hz, 1H, =C*H*), 5.91 (dt, *J* = 18.6, 1.0 Hz, 1H, *H*C=), 4.81–4.75 (m, 1H, *H*C-OH), 2.61 (t, *J* = 6.7 Hz, 2H, C*H*_2_), 2.00 (br s, 1H, O*H*), 0.32 (s, 3H, (Si-C*H_3_*)_2_). ^13^C-NMR (101 MHz, CDCl_3_) δ 144.4 (=CH), 144.0 (C), 138.8 (C), 133.9 (CH), 132.7 (HC=), 129.1 (CH), 128.5 (CH), 127.9 (CH), 127.7 (CH), 126.0 (CH), 73.5 (HC-OH), 47.0 (CH_2_), −2.4 ((Si-CH_3_)_2_).

### 3.5. TMSOTf-Promoted Cyclization Reaction to Afford Dihydropyranes **4**

A solution of 73 mg (0.33 mmol, 1.0 equiv.) of the E-vinylsilyl alcohol **3a** and the corresponding aldehyde (0.40 mmol, 1.2 equiv.), in 7 mL dichloromethane (0.05 M), was cooled to −78 °C under a nitrogen atmosphere. Then, TMSOTf (0.33 mmol, 1.0 equiv.) was added dropwise. The mixture was stirred at this temperature for 1 or 2 h while monitored by TLC. When the starting materials had been consumed, it was hydrolyzed with 6 mL of saturated NaHCO_3_. The phases were then separated, extracting the aqueous phase three times with dichloromethane. The organic phases were combined, washed with NaCl sat., and dried over anhydrous Na_2_SO_4_. The solvent was then evaporated under reduced pressure. The crude mixture was analyzed by NMR and then purified by column chromatography in silica gel, using mixtures of hexane-ethyl acetate, yielding dihydropyrans **4.**

(2R*,6S*)-6-benzyl-2-methyl-3,6-dihydro-2H-pyran (**4a**) was obtained following the general procedure 3.5, as a yellow oil, in 48% chemical yield (30 mg from 0.33 mmol of **3a**). ^1^H-NMR (500 MHz, CDCl_3_) δ 7.30–7.27 (m, 2H, Ar-H), 7.25–7.20 (m, 3H, Ar-H), 5.80–5.76 (m, 1H, HC=), 5.62–5.57 (m, 1H, =CH), 4.38–4.32 (m, 1H, O-CH), 3.73–3.66 (m, 1H, HC-O), 3.01 (dd, J = 13.5, 6.3 Hz, 1H, Ph-CHH), 2.68 (dd, J = 13.5, 8.1 Hz, 1H, Ph-CHH), 1.98–1.93 (m, 2H, CH_2_), 1.25 (d, J = 6.2 Hz, 3H, CH_3_). ^13^C-NMR (101 MHz, CDCl_3_) δ 138.4 (C), 129.8 (CH), 129.2 (=CH), 128.3 (CH), 126.3 (CH), 125.1 (HC=), 75.9 (O-CH), 70.2 (CH-O), 42.3 (Ph-CH_2_), 30.8 (CH_2_), 21.5 (CH_3_). HRMS (ESI^+^) m/z calc. for C_13_H_16_NaO ([M+Na]^+^): 211.1093, found 211.1097 [13].

(2R*,6S*)-2-methyl-6-phenethyl-3,6-dihydro-2H-pyran (**4b**) was obtained following the general procedure 3.5, as a yellow oil, in 45% chemical yield (30 mg from 0.33 mmol of **3a**). ^1^H-NMR (500 MHz, CDCl_3_) δ 7.30–7.27 (m, 2H, Ar-H), 7.23–7.15 (m, 3H, Ar-H), 5.84–5.78 (m, 1H, HC=), 5.65–5.61 (m, 1H, =CH), 4.17–4.10 (m, 1H, O-CH), 3.73–3.64 (m, 1H, HC-O), 2.80–2.70 (m, 2H, CH_2_-CH_2_-Ph), 2.00–1.94 (m, 2H, CH_2_), 1.87–1.80 (m, 2H, CH_2_-CH_2_-Ph), 1.26 (d, J = 5.8 Hz, 3H, CH_3_). ^13^C-NMR (101 MHz, CDCl_3_) δ 142.5 (C), 130.2 (CH), 128.7 (=CH), 128.4 (CH), 126.3 (CH), 125.2 (HC=), 74.1 (O-CH), 70.1 (CH-O), 37.3 (CH_2_-CH_2_-Ph), 33.1 (CH_2_), 31.4 (CH_2_-CH_2_-Ph), 21.9 (CH_3_). HRMS (ESI^+^) m/z calc. for C_14_H_18_NaO ([M+Na]^+^): 225.1250, found 225.1254. [32].

(2S*,6S*)-2-benzyl-6-methyl-3,6-dihydro-2H-pyran (**4c**) was obtained following the general procedure 3.5, as a yellow oil, in 48% chemical yield (22 mg from 0.24 mmol of **3b**). ^1^H-NMR (500 MHz, CDCl_3_) δ 7.31–7.26 (m, 2H, Ar-H), 7.25–7.18 (m, 3H, Ar-H), 5.76–5.70 (m, 1H, HC=), 5.63–5.56 (m, 1H, =CH), 4.28–4.20 (m, 1H, O-CH), 3.82–3.75 (m, 1H, HC-O), 3.01 (dd, J = 13.6, 6.5 Hz, 1H, Ph-CHH), 2.69 (dd, J = 13.6, 7.0 Hz, 1H, Ph-CHH), 2.07–1.96 (m, 1H, CHH), 1.90–1.81 (m, 1H, CHH), 1.24 (d, J = 6.8 Hz, 3H, CH_3_). ^13^C-NMR (101 MHz, CDCl_3_) δ 138.7 (C), 131.6 (=CH), 129.5 (CH), 128.3 (CH), 126.3 (CH), 124.1 (HC=), 75.1 (CH-O), 71.3 (O-CH), 42.7 (Ph-CH_2_), 33.0 (CH_2_), 21.5 (CH_3_). HRMS (ESI^+^) m/z calc. for C_13_H_16_NaO ([M+Na]^+^): 211.1093, found 211.1097.

(2S*,6S*)-2-benzyl-6-cyclohexyl-3,6-dihydro-2H-pyran (**4d**) was obtained following the general procedure 3.5, as a yellow oil, in 80% chemical yield (49 mg from 0.24 mmol of **3b**). ^1^H-NMR (500 MHz, CDCl_3_) δ 7.32–7.17 (m, 5H, Ar-H), 5.81–5.73 (m, 1H, HC=), 5.68–5.62 (m, 1H, =CH), 3.92–3.85 (m, 1H, O-CH), 3.77–3.68 (m, 1H, HC-O), 2.95 (dd, J = 13.7, 6.8 Hz, 1H, Ph-CHH), 2.68 (dd, J = 13.7, 6.1 Hz, 1H, Ph-CHH), 2.05–1.93 (m, 1H, CHH_cycle_), 1.90–1.82 (m, 1H, CHH_cycle_), 1.82–1.61 (m, 5H, CH_2_), 1.50–1.39 (m, 1H, CH), 1.31–1.01 (m, 5H, CH_2c_). ^13^C-NMR (101 MHz, CDCl_3_) δ 139.1 (C), 129.6 (CH), 129.1 (=CH), 128.2 (CH), 126.1 (CH), 125.0 (HC=), 79.2 (O-CH), 74.8 (HC-O), 42.9 (CH), 42.7 (CH_2_-Ph), 31.2 (CH_2cycle_), 28.8 (CH_2_), 28.3 (CH_2_), 26.8 (CH_2_), 26.5 (CH_2_), 26.5 (CH_2_). HRMS (ESI^+^) m/z calc. for C_18_H_24_NaO ([M+Na]^+^): 279.1719, found 279.1722.

(2S*,6S*)-2-benzyl-6-propyl-3,6-dihydro-2H-pyran (**4e**) was obtained following the general procedure 3.5, as a yellow oil, in 60% chemical yield (31 mg from 0.24 mmol of **3b**). ^1^H RMN (500 MHz, CDCl_3_) δ 7.32–7.18 (m, 5H, Ar-H), 5.79–5.71 (m, 1H, HC=), 5.65–5.58 (m, 1H, =CH), 4.13–4.04 (m, 1H, O-CH), 3.80–3.70 (m, 1H, HC-O), 2.98 (dd, J = 13.7, 6.9 Hz, 1H, Ph-CHH), 2.70 (dd, J = 13.7, 6.2 Hz, 1H, Ph-CHH), 2.10–1.98 (m, 1H, CHH), 1.92–1.82 (m, 1H, CHH), 1.60–1.37 (m, 4H, CH_2_CH_2_), 0.92 (t, J = 6.9 Hz, 3H, CH_3_). ^13^C-NMR (101 MHz, CDCl_3_) δ 139.0 (C), 130.8 (=CH), 129.6 (CH), 128.3 (CH), 126.2 (CH), 124.5 (HC=), 75.0 (HC-O), 74.9 (O-CH), 42.6 (Ar-CH_2_), 37.9 (CH_2_), 31.2 (CH_2_), 18.6 (CH_2_), 14.2 (CH_3_). HRMS (ESI^+^) m/z calc. for C_15_H_20_NaO ([M+Na]^+^): 239.1406, found 239.1410.

(2S*,6S*)-2-benzyl-6-phenethyl-3,6-dihydro-2H-pyran (**4f**) was obtained following the general procedure 3.5, as a yellow oil, in 79% chemical yield (53 mg from 0.24 mmol of **3b**). ^1^H-NMR (500 MHz, CDCl_3_) δ 7.34–7.28 (m, 4H, Ar-*H*), 7.25–7.20 (m, 3H, Ar-*H*), 7.17–7.13 (m, 1H, Ar-*H*), 7.07–7.04 (m, 2H, Ar-*H*), 5.80–5.75 (m, 1H, *H*C=), 5.61–5.56 (m, 1H, =C*H*), 4.02–3.97 (m, 1H, O-C*H*), 3.78–3.72 (m, 1H, *H*C-O), 2.96 (dd, *J* = 13.7, 7.8 Hz, 1H, Ph-C*H*H), 2.80–2.67 (m, 3H, Ph-CH*H*; C*H_2_*-CH_2_-Ph), 2.12–2.04 (m, 1H, C*H*H), 1.97–1.90 (m, 1H, CH*H*), 1.87–1.73 (m, 2H, C*H_2_*-Ph). ^13^C-NMR (101 MHz, CDCl_3_) δ 142.3 (C), 139.1 (C), 130.6 (=CH), 129.6 (CH), 128.8 (CH), 128.4 (CH), 128.3 (CH), 126.3 (CH), 125.7 (CH), 124.9 (HC=), 75.1 (CH-O), 73.7 (O-CH), 42.7 (Ph-*C*H_2_), 37.3 (*CH_2_-*Ph), 31.4 (*CH_2_*-CH_2_-Ph), 31.3 (*C*H_2_). HRMS (ESI^+^) m/z calc. for C_20_H_22_NaO ([M+Na]^+^): 301.1563, found 301.1564.

4-(2-((2S*,6S*)-6-benzyl-5,6-dihydro-2H-pyran-2-yl)ethyl)phenol (**4g**) was obtained following the general procedure 3.5, as a yellow oil, in 43% chemical yield (30 mg from 0.24 mmol of **3b**). ^1^H-NMR (500 MHz, CDCl_3_) δ 7.34–7.27 (m, 5H, Ar-*H*), 7.25–7.21 (m, 1H, Ar-*H*), 6.88 (d, *J* = 8.6 Hz, 2H, Ar-*H*), 6.67 (d, *J* = 8.6 Hz, 2H, Ar-*H*), 5.80–5.73 (m, 1H, *H*C=), 5.60–5.53 (m, 1H, =C*H*), 4.49 (s, 1H, O*H*), 3.99–3.91 (m, 1H, O-C*H*), 3.77–3.70 (m, 1H, *H*C-O), 2.94 (dd, *J* = 13.4, 7.3 Hz, 1H, Ph-CHH), 2.76 (dd, *J* = 13.4, 5.8 Hz, 1H, Ph-CHH), 2.71–2.59 (m, 2H, CH_2_-C*H*_2_-Ar), 2.12–2.02 (m, 1H, C*H*H), 1.98–1.90 (m, 1H, CH*H*), 1.83–1.74 (m, 1H, C*H*H-CH_2_-Ar), 1.73–1.65 (m, 1H, CH*H*-CH_2_-Ar). ^13^C-NMR (101 MHz, CDCl_3_) δ 153.5 (C), 139.2 (C), 134.5 (C), 130.6 (=CH), 129.9 (CH), 129.6 (CH), 128.3 (CH), 126.3 (CH), 124.8 (HC=), 115.1 (CH), 75.1 (CH-O), 73.6 (O-CH), 42.7 (Ph-CH_2_), 37.5 (*CH_2_*-CH_2_-Ar), 31.3 (CH_2_), 30.4 (CH_2_-*CH_2_*-Ar). HRMS (ESI^+^) m/z calc. for C_20_H_22_NaO_2_ ([M+Na]^+^): 317.1512, found 317.1513.

((2R*,6S*)-6-benzyl-5,6-dihydro-2H-pyran-2-yl)methyl propionate (**4h**) was obtained following the general procedure 3.5, as a yellow oil, in 46% chemical yield (30 mg from 0.24 mmol of **3b**). ^1^H-NMR (500 MHz, CDCl_3_) δ 8 7.31–7.17 (m, 5H, Ar-*H*), 5.92–5.85 (m, 1H, *H*C=), 5.63–5.57 (m, 1H, =C*H*), 4.39–4.32 (m, 1H, O-C*H*), 4.15 (dd, *J* = 11.4, 6.1 Hz, 2H, *H*HC-O), 4.11 (dd, *J* = 11.4, 4.8 Hz, 2H, H*H*C-O), 3.83–3.74 (m, 1H, *H*C-O), 2.78 (dd, *J* = 13.8, 7.0 Hz, 1H, Ph-C*H*H), 2.72 (dd, *J* = 13.8, 6.2 Hz, 1H, Ph-CH*H*), 2.62–2.54 (m, 1H, C*H*), 2.11–2.01 (m, 1H, C*H*H), 1.96–1.87 (m, 1H, CH*H*), 1.17 (d, *J* = 7.0 Hz, 3H, C*H_3_*), 1.16 (d, *J* = 7.0 Hz, 3H, C*H_3_*). ^13^C-NMR (101 MHz, CDCl_3_) δ 177.1 (C), 138.6 (C), 129.5 (CH), 128.3 (CH), 127.1 (HC=), 126.3 (=CH), 126.1 (CH), 74.8 (CH-O), 73.4 (O-CH), 66.2 (CH_2_-O), 42.4 (Ar-CH_2_), 34.1 (CH), 30.8 (CH_2_), 19.2 (CH_3_), 19.1 (CH_3_). HRMS (ESI^+^) m/z calc. for C_18_H_24_NaO ([M+Na]^+^): 279.1719, found 279.1722.

(2S*,6S*)-2,6-dibenzyl-3,6-dihydro-2H-pyran (**4i**) was obtained following the general procedure 3.5, as a yellow oil, in 37% chemical yield (23 mg from 0.24 mmol of **3b**). ^1^H-NMR (400 MHz, CDCl_3_) δ 7.30–7.27 (m, 2H, Ar-*H*), 7.25–7.17 (m, 8H, Ar-*H*), 5.79–5.74 (m, 1H, *H*C=), 5.64–5.60 (m, 1H, =C*H*), 4.35–4.28 (m, 1H, O-C*H*), 3.78–3.72 (m, 1H, *H*C-O), 2.99–2.92 (m, 2H, Ph-C*H*H + *H*HC-Ph), 2.76–2.67 (m, 2H, Ph-CH*H* + H*H*C-Ph), 2.09–2.00 (m, 1H, C*H*H_cycle_), 1.93–1.86 (m, 1H, CH*H*_cycle_). ^13^C-NMR (101 MHz, CDCl_3_) δ 138.8 (C), 138.6 (C), 129.7 (HC=), 129.6 (CH), 128.3 (CH), 126.3 (CH), 126.1 (CH), 125.1 (=CH), 76.0 (O-CH), 75.1 (HC-O), 42.6 (Ph-CH_2_), 42.2 (CH_2_-Ph), 31.0 (CH_2_). HRMS (ESI^+^) m/z calc. for C_19_H_20_NaO ([M+Na]^+^): 287.1406, found 287.1406 [33]. 

### 3.6. BF_3_·OEt_2_-Promoted Diels Alder Reaction to Afford Derivatives **5**

A solution of 60 mg (0.20 mmol, 1.0 equiv.) of protected allylsilyl alcohol **3a** and 30 μL trans-cinnamaldehyde (0.36 mmol, 1.2 equiv.), in 6 mL dichloromethane (0.05 M), was cooled to 0 °C under a nitrogen atmosphere. Then, BF_3_·OEt_2_ (0.36 mmol, 1.2 equiv.) was added dropwise. The mixture was stirred at this temperature for 4 h while being monitored by TLC. When the starting materials had been consumed, it was hydrolyzed with 5 mL of saturated NaHCO_3_. The phases were then separated, extracting the aqueous phase three times with dichloromethane. The organic phases were combined, washed with NaCl sat., and dried over anhydrous Na_2_SO_4_. The solvent was then evaporated under reduced pressure. The crude mixture was analyzed by NMR and then purified by column chromatography in silica gel, using a mixture of hexane-ethyl acetate (20:1), affording aldehyde **5**.

*3-methyl-1,2,3,6-tetrahydro-[1,1’-biphenyl]-2-carbaldehyde* (**5**) was obtained as a yellow oil, in 43% chemical yield (13 mg from 0.20 mmol of **3a**). ^1^H-NMR (400 MHz, CDCl_3_) δ 9.62 (d, J = 3.8 Hz, 1H, CHO), 7.33–7.28 (m, 2H, Ar-H), 7.25–7.20 (m, 1H, Ar-H), 5.83–5.72 (m, 2H, HC=CH), 3.36–3.28 (m, 1H, HC-Ph), 2.84–2.77 (m, 1H, HC-CHO), 2.68 2.57 (m, 1H, HC-CH_3_), 2.48–2.37 (m, 1H, CHH), 2.27–2.17 (m, 1H, CHH), 1.10 (d, J = 6.7 Hz, 3H, CH_3_). ^13^C-NMR (101 MHz, CDCl_3_) δ 206.1 (CHO), 144.0 (C), 131.9 (=CH), 128.9 (CH), 127.8 (CH), 126.8 (CH), 126.0 (HC=), 55.1 (HC-CHO), 37.0 (HC-Ph), 32.8 (CH_2_), 29.9 (HC-CH_3_), 17.0 (CH_3_). HRMS (ESI+) m/z calc. for C_14_H_15_O ([M-H]^−^): 199.1128, found 199.1124.

## 4. Conclusions

Allyl(phenyldimethyl)silane **1** (obtained in a quantitative synthesis without any further purification steps) is a versatile substrate that has been used to prepare, in a regioselective manner, either allyl- or vinylsilyl alcohols, choosing the appropriate reaction protocols. Thus, *E*-vinylsilyl alcohols were suitable coupling agents in silyl-Prins cyclizations, with highly reactive alkyl aldehydes in the presence of TMSOTf. These Prins cyclization reactions allowed the preparation of a battery of *cis*-2,6-disubstituted dihydropyrane derivatives, in moderate to good yields, and with good to excellent diastereoselectivities (>90:10). The cyclization reaction turned out to be very sensitive to different reaction conditions, particularly for allylsilyl alcohols and non-activated aldehydes, in which we could identify and isolate side-products resulting from oxonia-Cope [34] transposition and Peterson elimination processes. Computational studies showed a very low energy profile for the cyclization of the oxygenated heterocycle, indicating that such cyclization is a reversible process from a thermodynamic point of view. 

## Figures and Tables

**Table 1:**
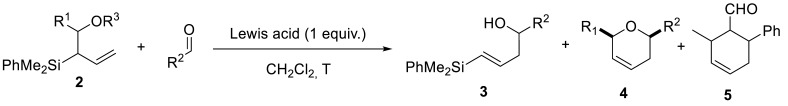
Screening of the reaction conditions.

Entry	R^1^	R^2^	R^3^	LA (1 equiv.)	T (°C)	NMR Analysis (Isolated Yield)
1	CH_3_	CH_2_CH_2_Ph	H	TMSOTf	−78	**3**
2	CH_3_	CH_2_CH_2_Ph	H	TMSOTf	0	Complex mixture
3 ^a^	CH_3_	CH_2_CH_2_Ph	H	TMSOTf	−78	Complex mixture
4	CH_3_	CH_2_CH_2_Ph	H	BF_3_·OEt_2_	−78	Complex mixture
5	CH_3_	CH=CHPh	H	TMSOTf	−20	Aldehyde
6 ^a^	CH_2_Ph	CH_3_	H	TMSOTf	−78	**3a** + **4a**
7 ^a^	CH_2_Ph	CH_2_Ph	H	TMSOTf	−78	**4i**
8 ^a^	Ph	CH_3_	H	TMSOTf	−78	Complex mixture
9	Ph	CH_2_CH_2_Ph	H	TMSOTf	−78	Complex mixture
10	CH_3_	CH=CHPh	TMS	TMSOTf	−78	**2a**
11	CH_3_	CH=CHPh	TMS	BF_3_·OEt_2_	−78	Complex mixture
12	CH_3_	CH=CHPh	TMS	BF_3_·OEt_2_	0	**5** (29%)

^a^ With 3 equiv. of aldehyde instead of 1.2 equiv.

**Table 2:**
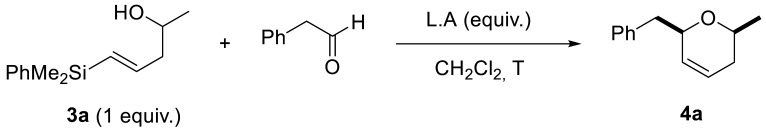
Screening of the reaction conditions.

Entry	LA (equiv.)	Temperature (°C)	Isolated Yield
1 ^a^	BF_3_·OEt_2_ (1.0)	0	Complex mixture
2	BF_3_·OEt_2_ (1.2)	0	Complex mixture
3	BF_3_·OEt_2_ (0.5)	0	Complex mixture
4	TMSCl (1.5)	0 to rt ^b^	nr
5 ^a^	TMSOTf (1.0)	−78	35%
6	TMSOTf (1.0)	−78	48%
7	TMSOTf (0.5)	−78	25%

^a^ With 3 equiv. of aldehyde. ^b^ Room temperature.

## Data Availability

All data can be found in supporting information.

## Supplementary Materials

The following supporting information can be downloaded at: https://www.mdpi.com/article/10.3390/molecules28073080/s1, with contains the ^1^H- and ^13^C-NMR spectra of the synthetized compounds, and Cartesian coordinates for theoretical studies. Figure S1. Relative energies for Reactants and products for *S* isomer and *R* isomer respectively computed at BP86-D3 triple-z theoretical level. Scheme S1. Sequence of consecutive rotations for sigma bonds that place bulky groups in equatorial arrangement for *R* isomer.
