# Supplementary material for: Diastereoselective Synthesis of cis-2,6-Disubstituted Dihydropyrane Derivatives through a Competitive Silyl-Prins Cyclization versus Alternative Reaction Pathways
Source: Molecules. 2023 Mar 30;28(7):3080. doi: 10.3390/molecules28073080 (PMC10096261; doi:10.3390/molecules28073080)
Supplement: Supplementary file 1 [file molecules-28-03080-s001.zip › molecules-2264742-supplementary.pdf]

SUPPLEMENTARY MATERIALS

Diastereoselective synthesis of *cis*-2,6-disubstituted dihydropyrane derivatives through a competitive silyl-Prins cyclization versus alternative reaction pathways

Laura F. Peña[1], Enol López[1], Ángel Sánchez-González[1,2] and Asunción Barbero[1*]

[1] Department of Organic Chemistry, Campus Miguel Delibes, University of Valladolid, 47011, Valladolid, Spain.
[2] BioISI-Biosystems and Integrative Sciences Institute, Departamento de Química e Bioquímica, Faculdade de Ciências, Universidade de Lisboa, Campo Grande, 1749-016 Lisboa (Portugal)

- Correspondence: asuncion.barbero@uva.es

Table of Contents

1. Copies of NMR Spectra S-2
2. Cartesians Coordinates S-20

COPIES OF NMR SPECTRA

Compound 2a

^{1}H NMR (500 MHz, $CDCl_3$)

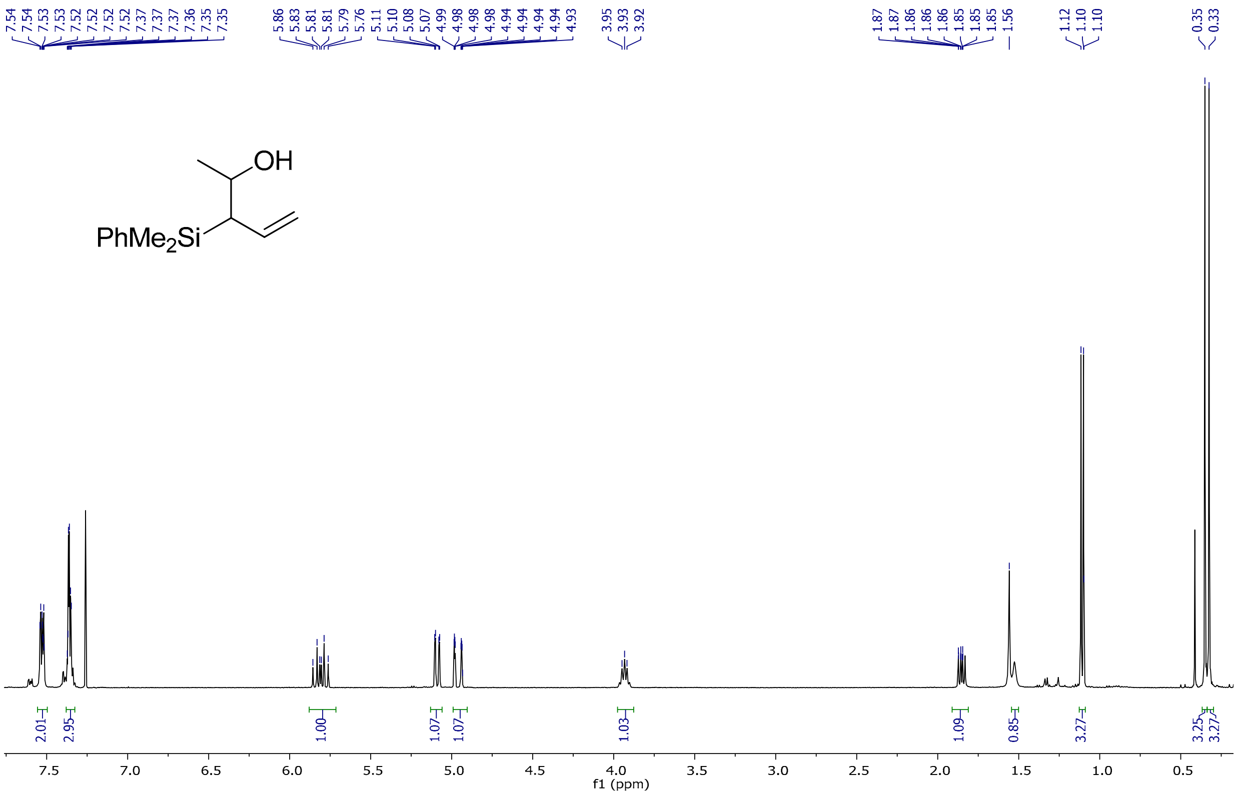

^{13}C NMR (101 MHz, $CDCl_3$)

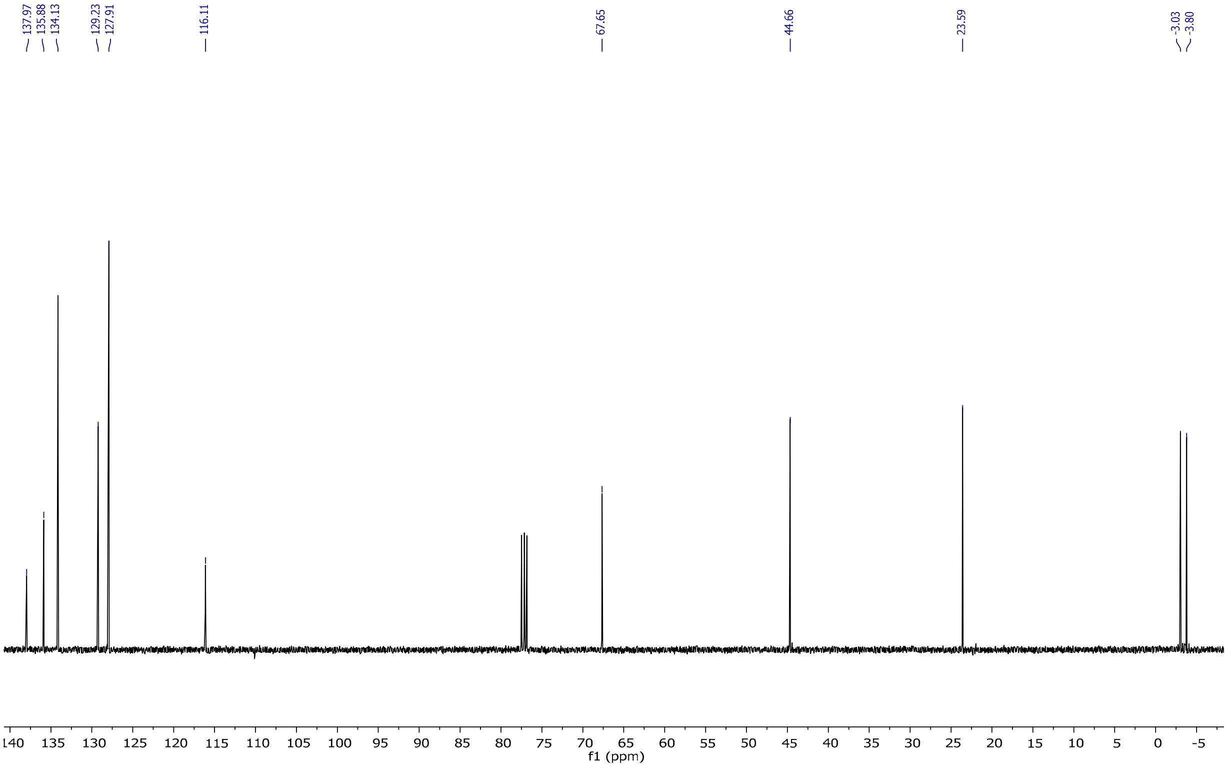

Compound 2b

¹H NMR (500 MHz, $CDCl_3$)

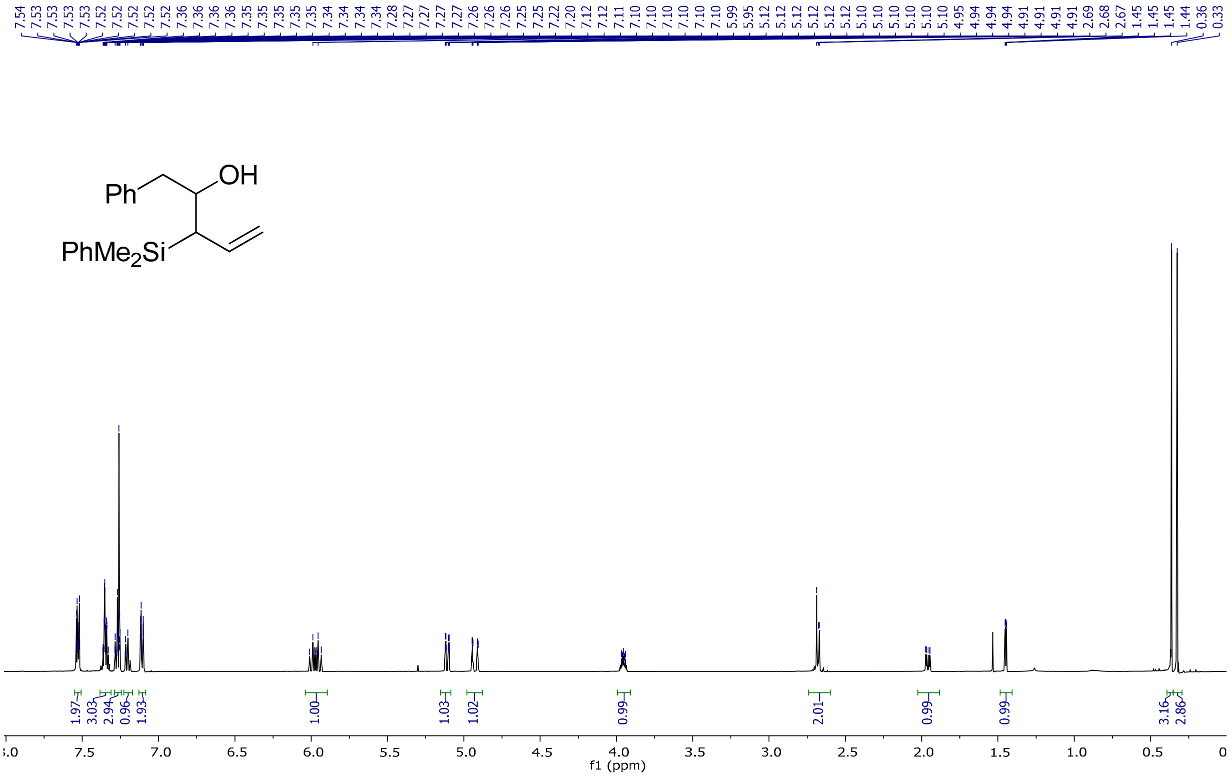

¹³C NMR (101 MHz, $CDCl_3$)

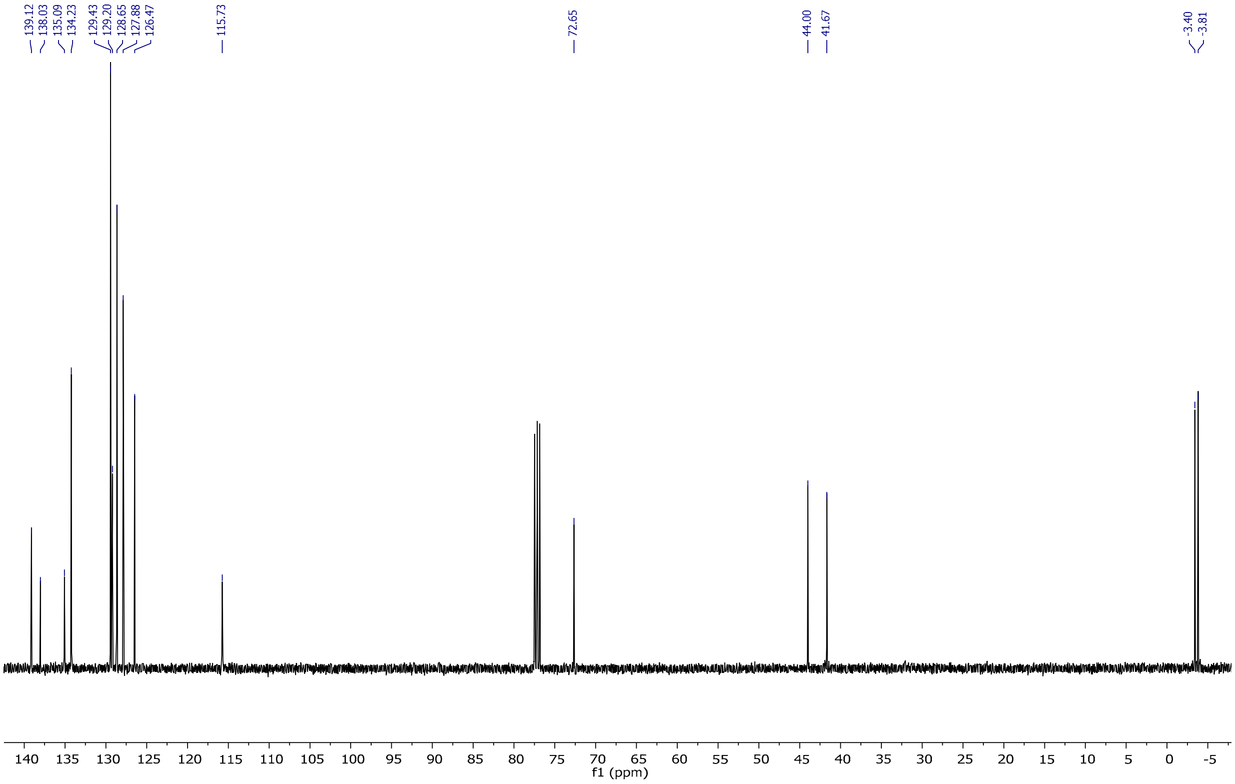

Compound 2c

¹H NMR (500 MHz, $CDCl_3$)

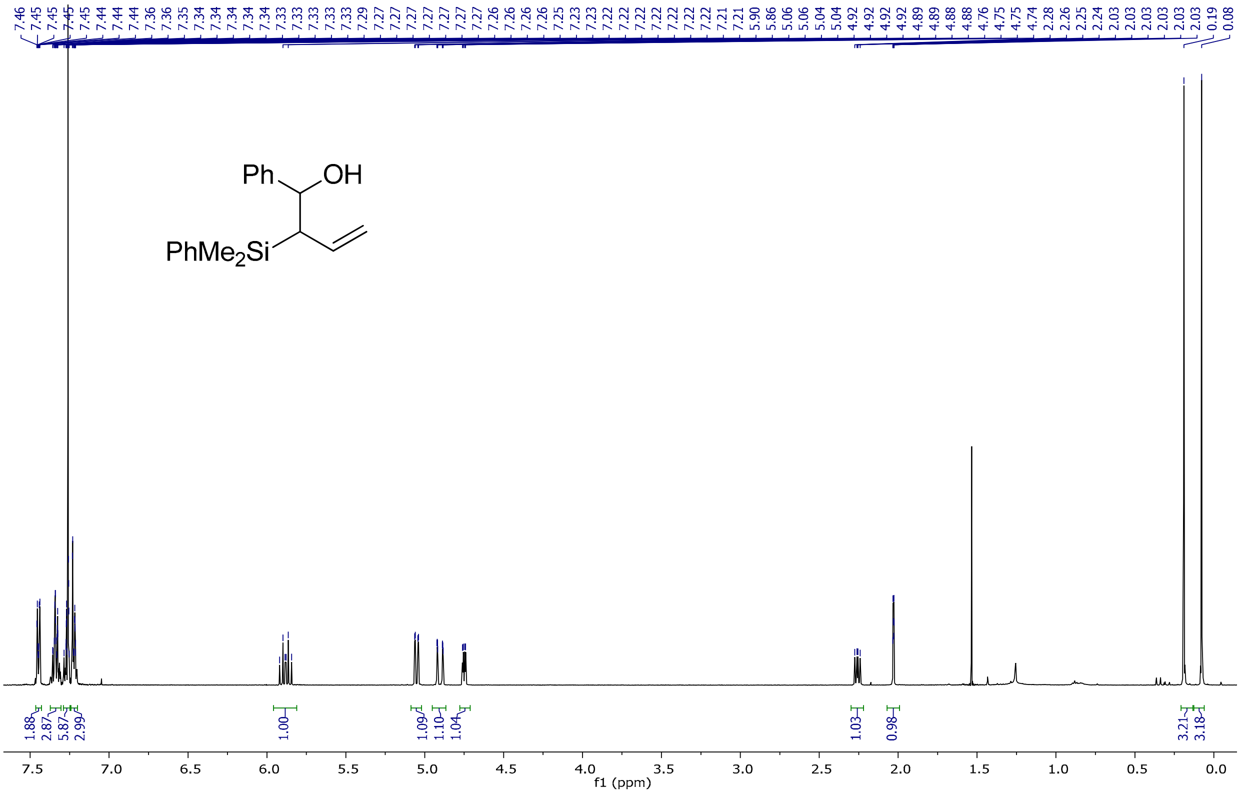

¹³C NMR (101 MHz, $CDCl_3$)

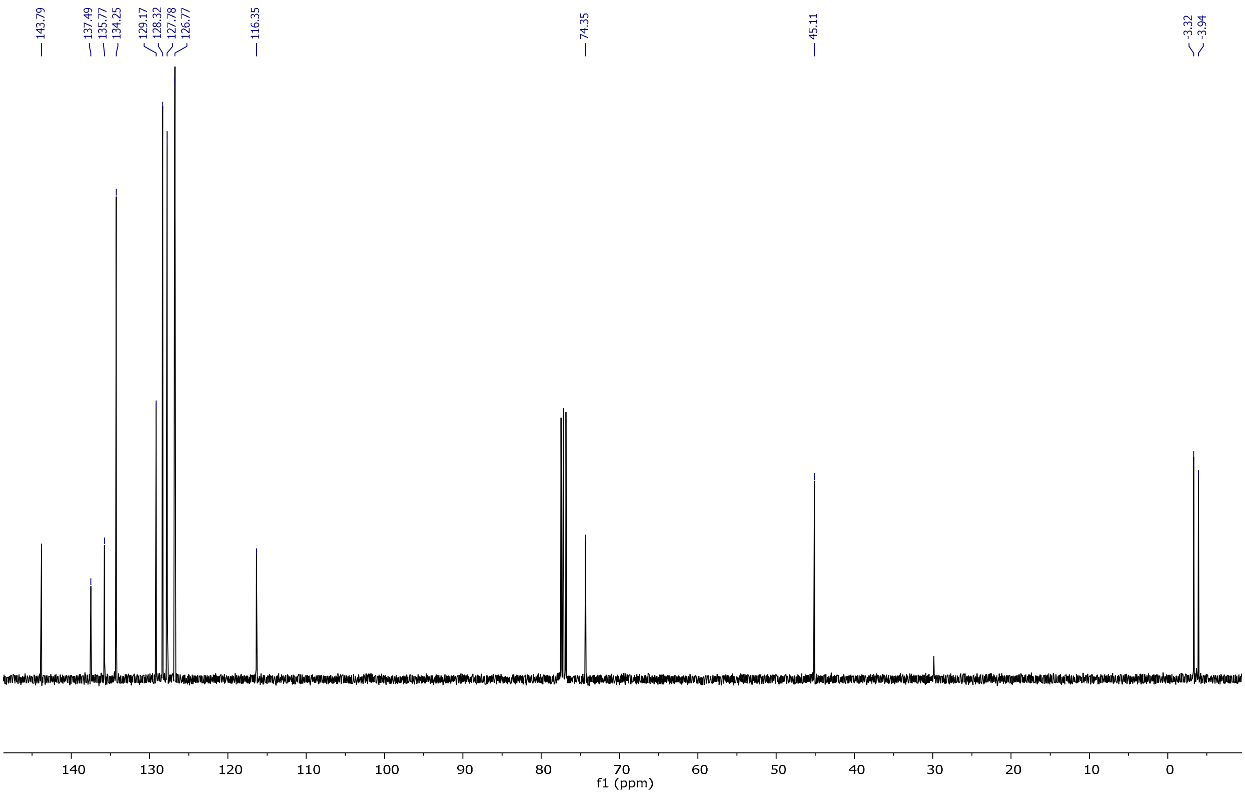

Compound 3a

^{1}H NMR (500 MHz, $CDCl_3$)

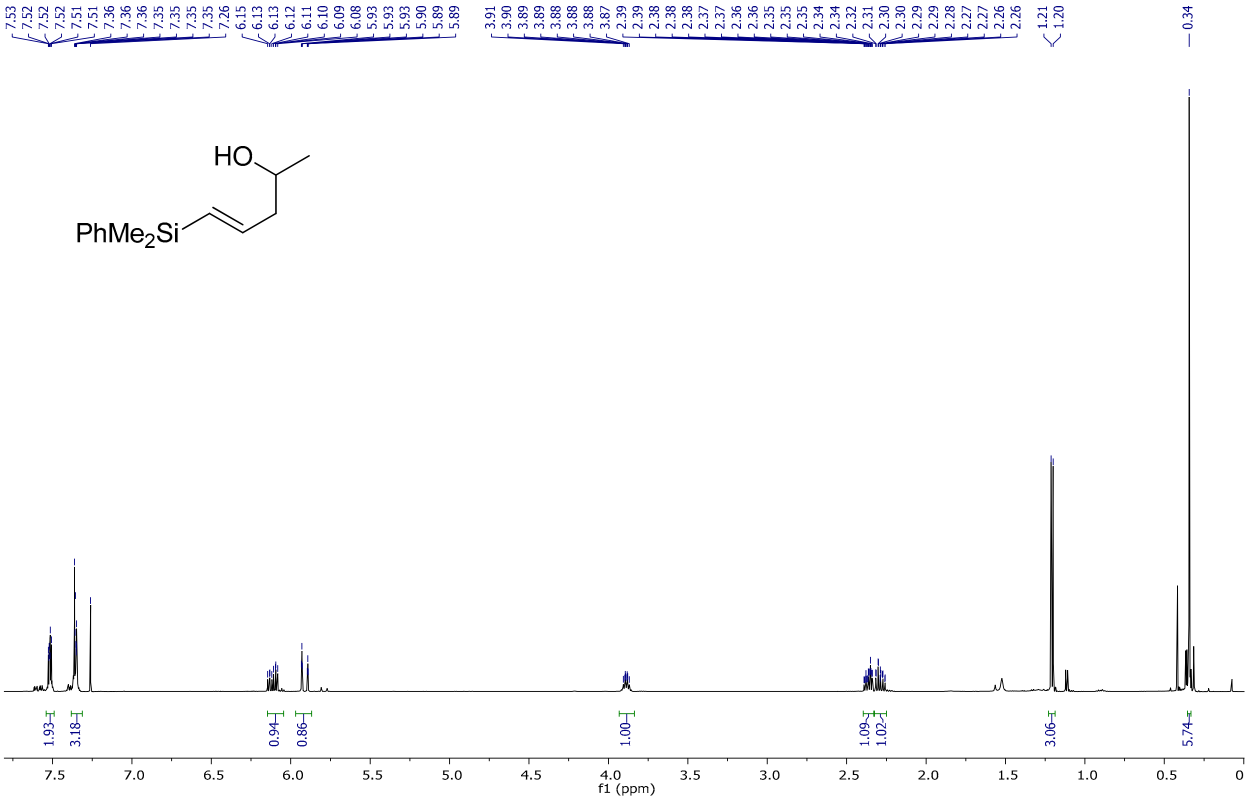

^{13}C NMR (101 MHz, $CDCl_3$)

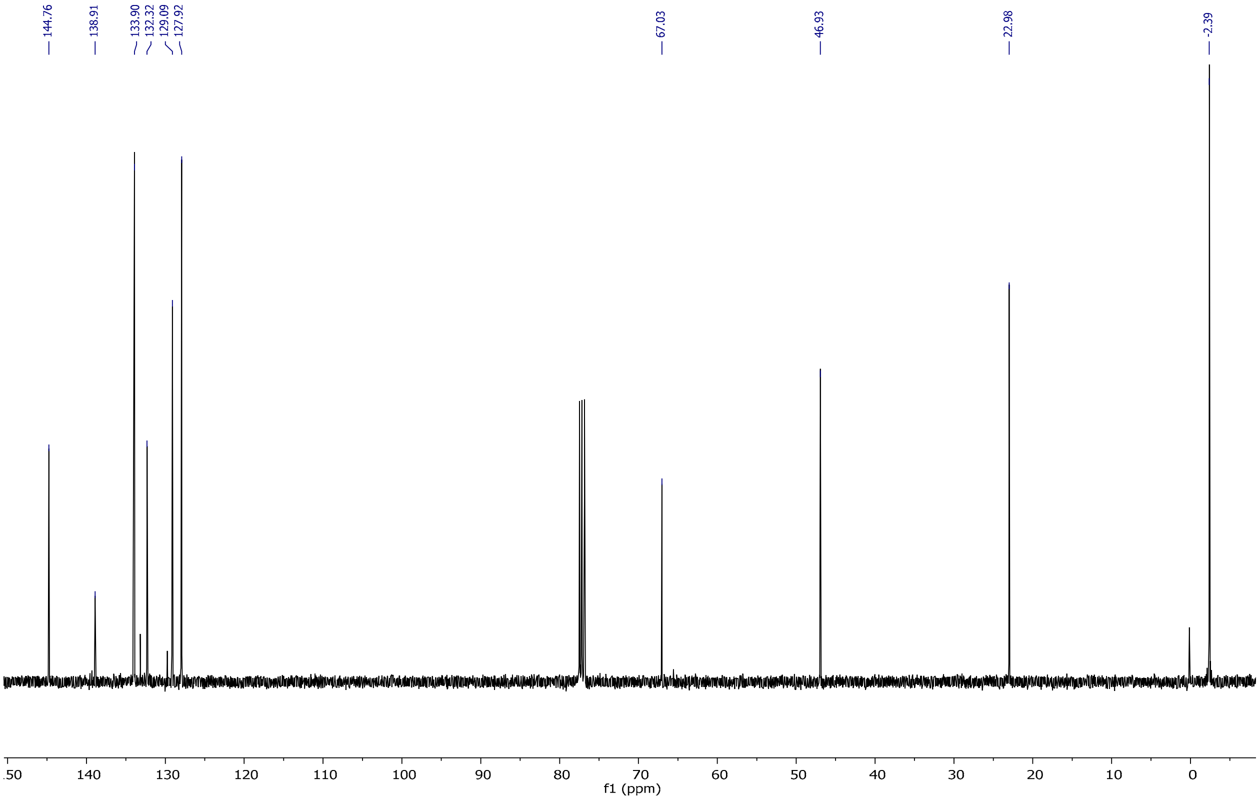

Compound 3b

^{1}H NMR (500 MHz, $CDCl_3$)

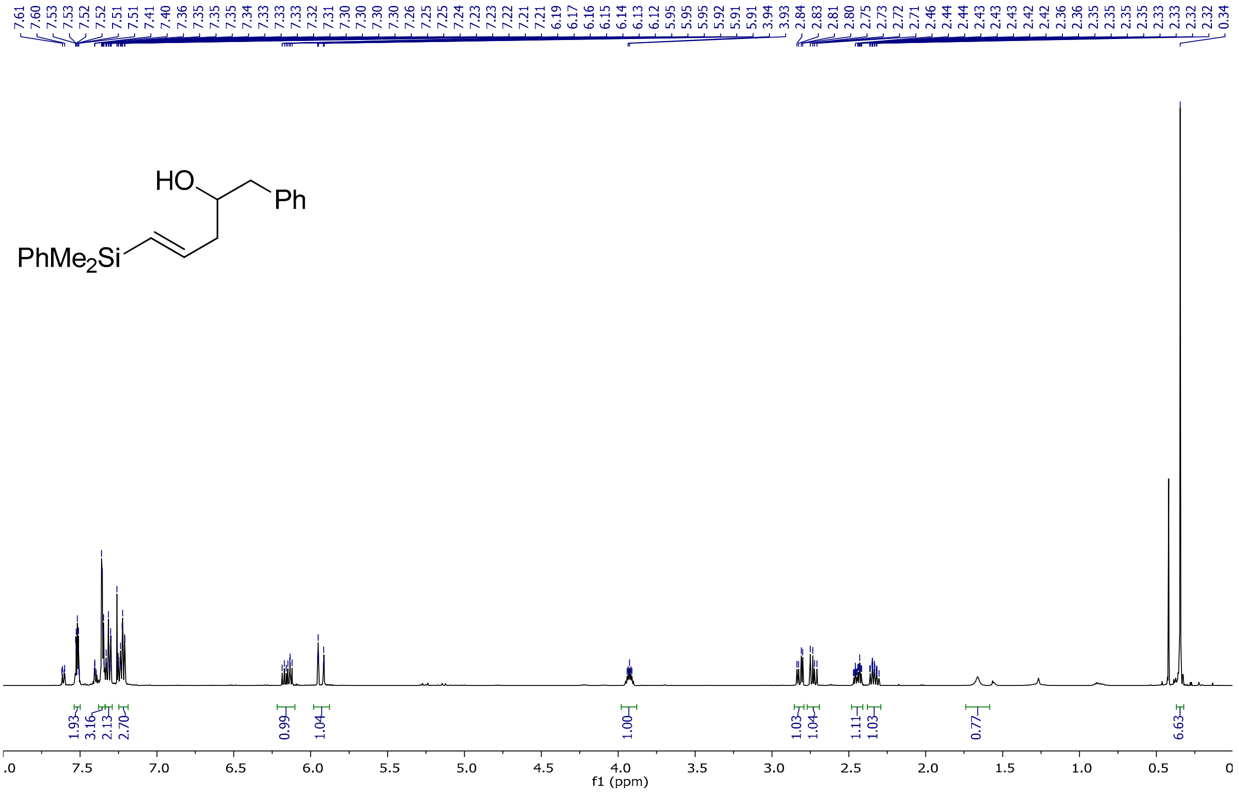

^{13}C NMR (101 MHz, $CDCl_3$)

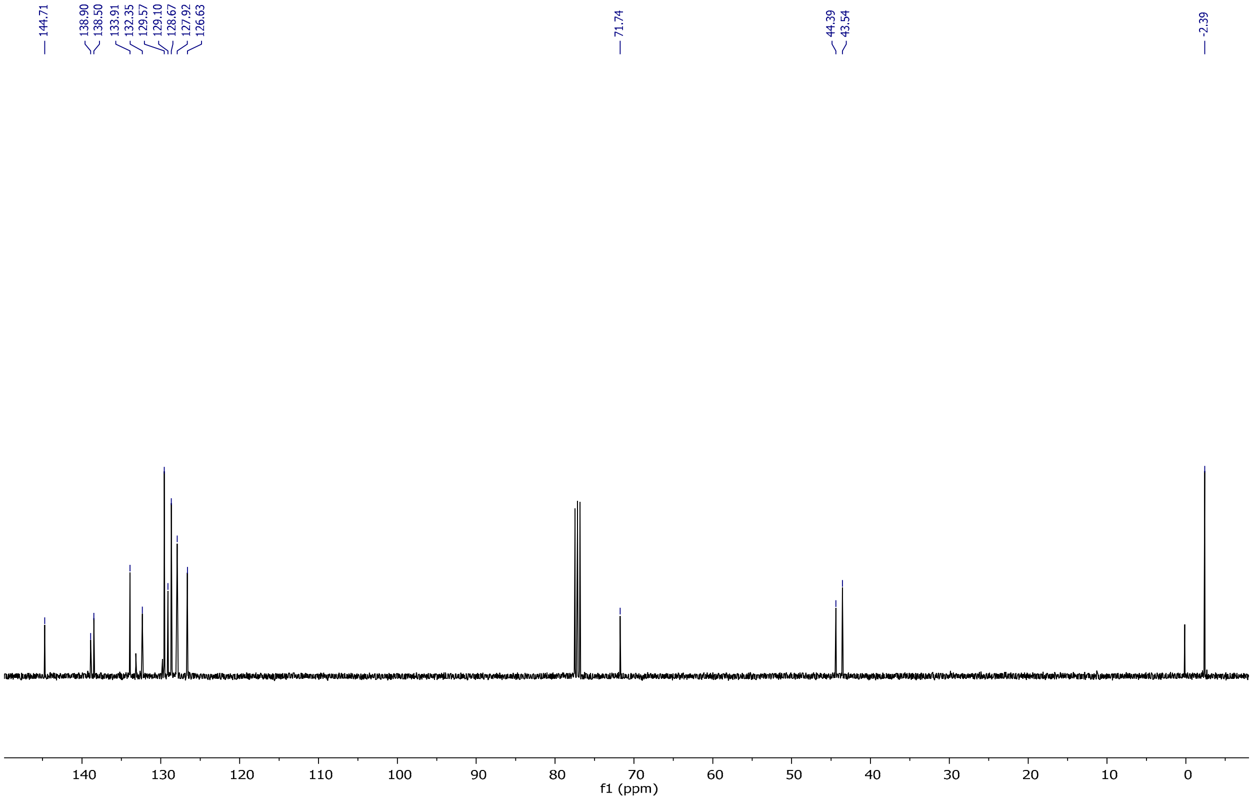

Compound 3c

^{1}H NMR (500 MHz, $CDCl_3$)

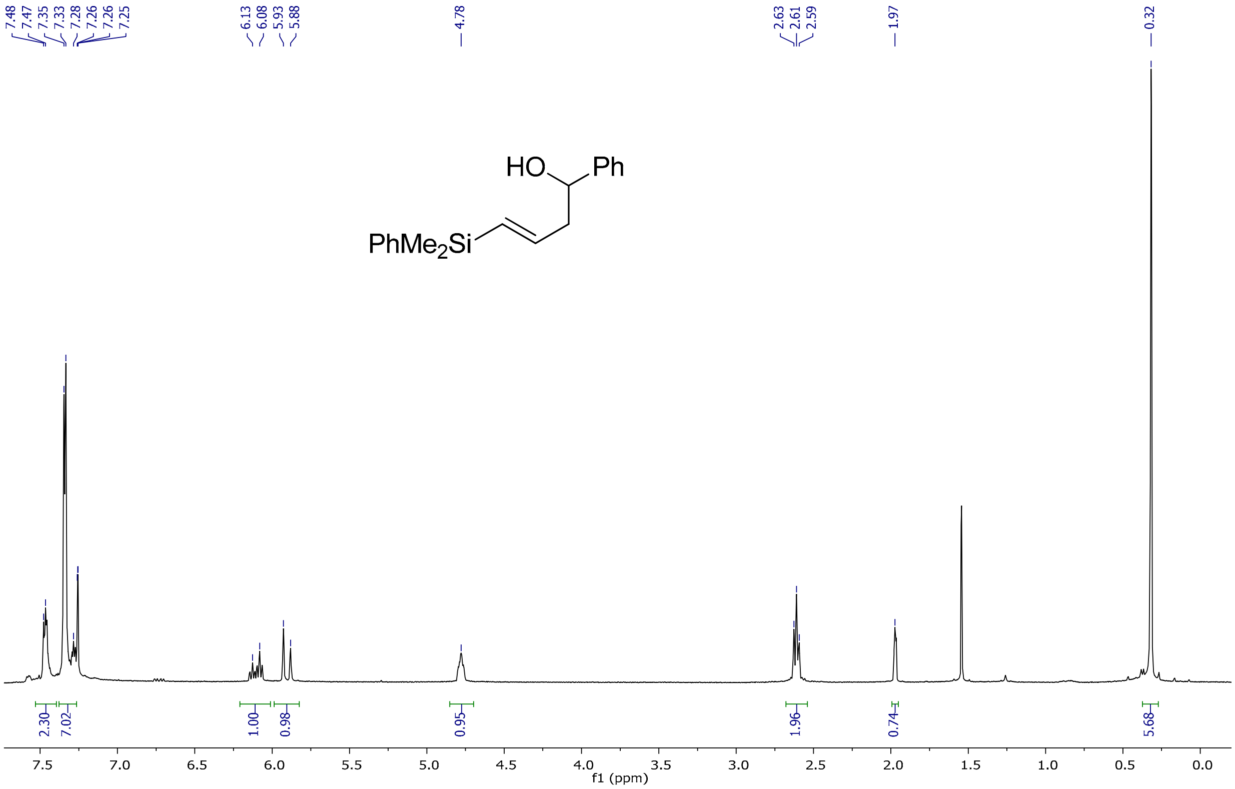

^{13}C NMR (101 MHz, $CDCl_3$)

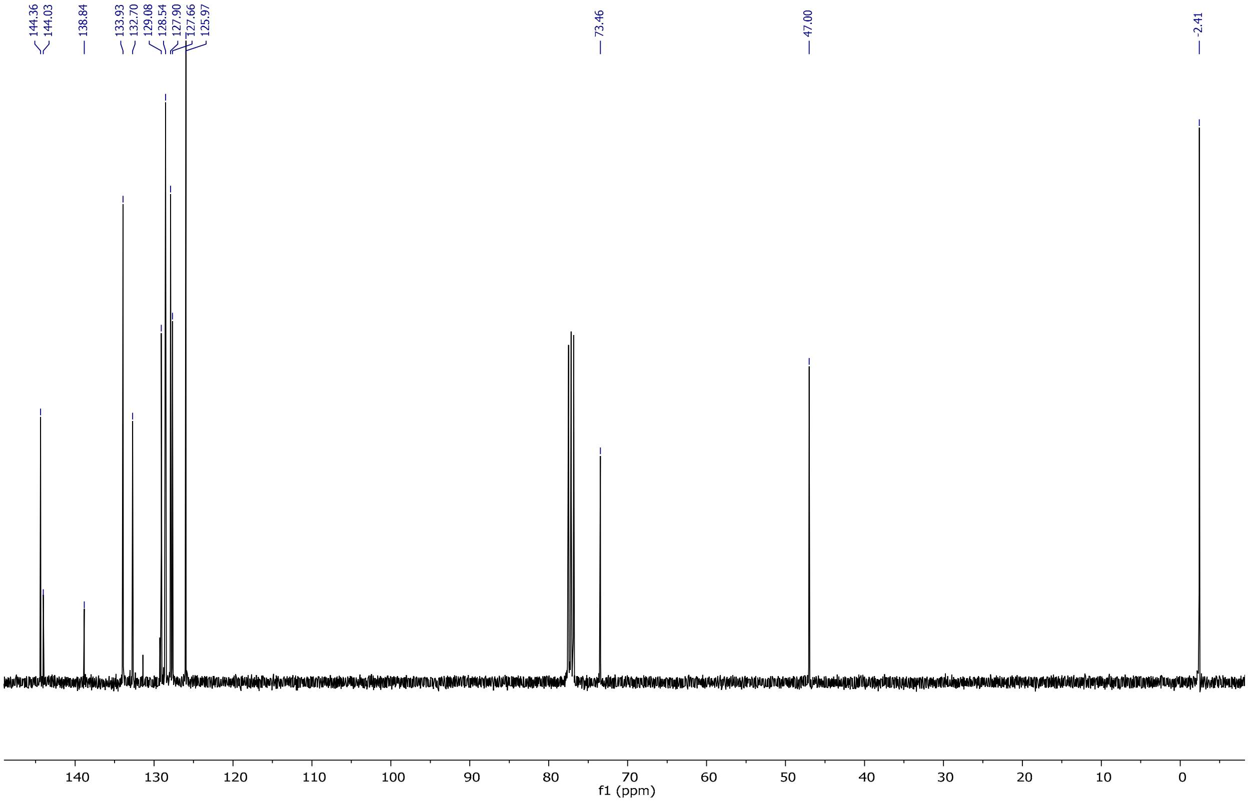

Compound 4a

^{1}H NMR (500 MHz, $CDCl_3$)

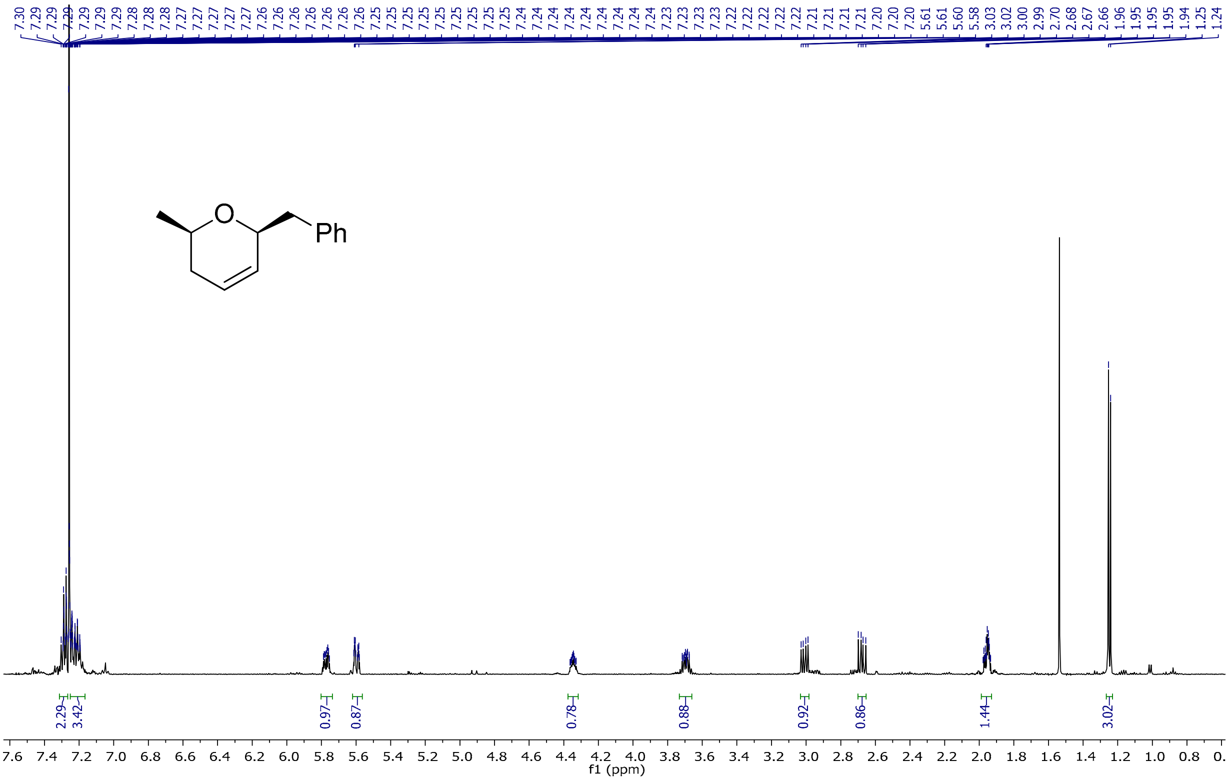

^{13}C NMR (101 MHz, $CDCl_3$)

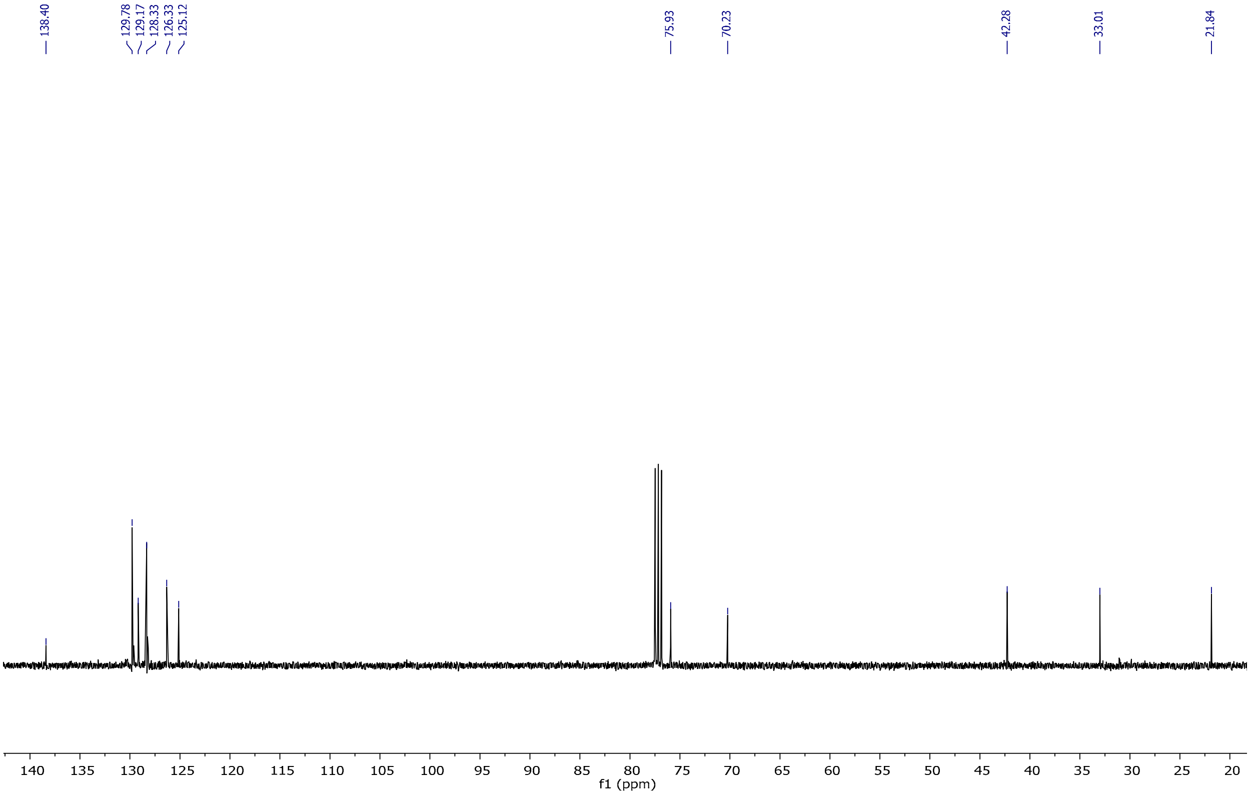

COSY

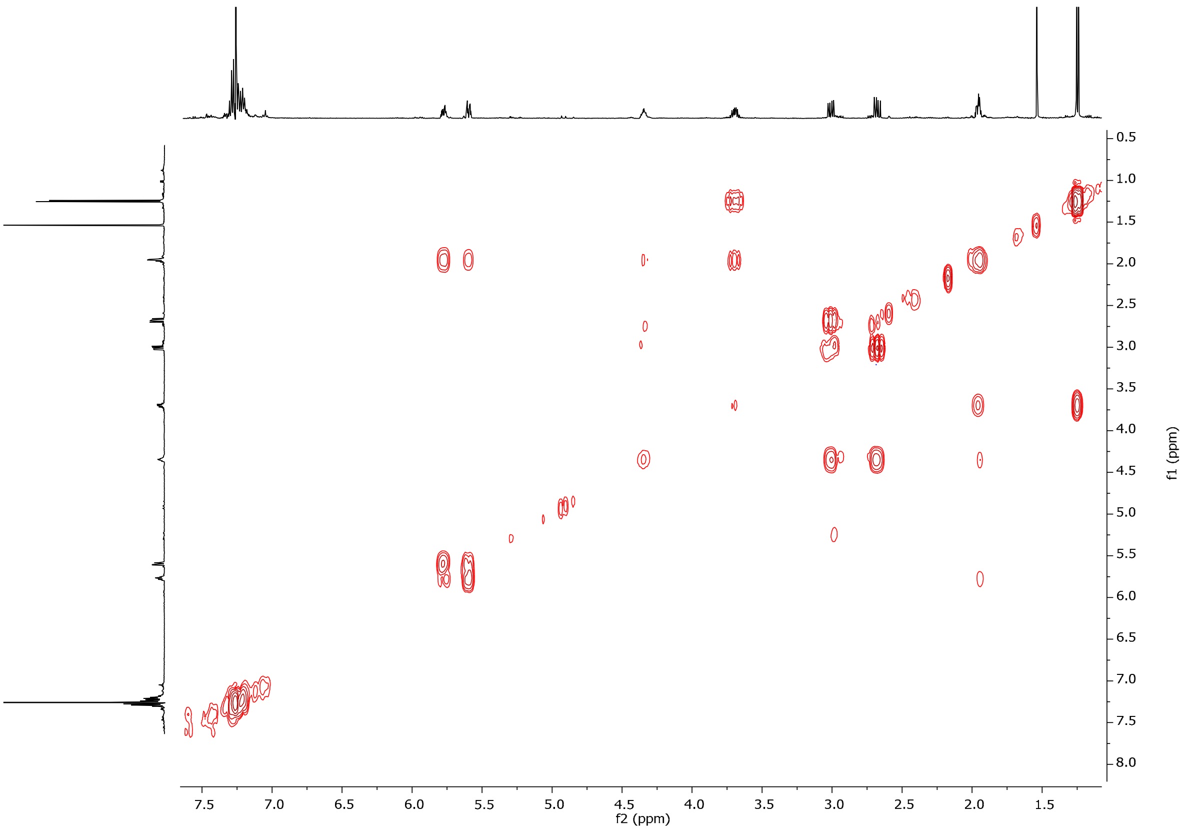

HSQC

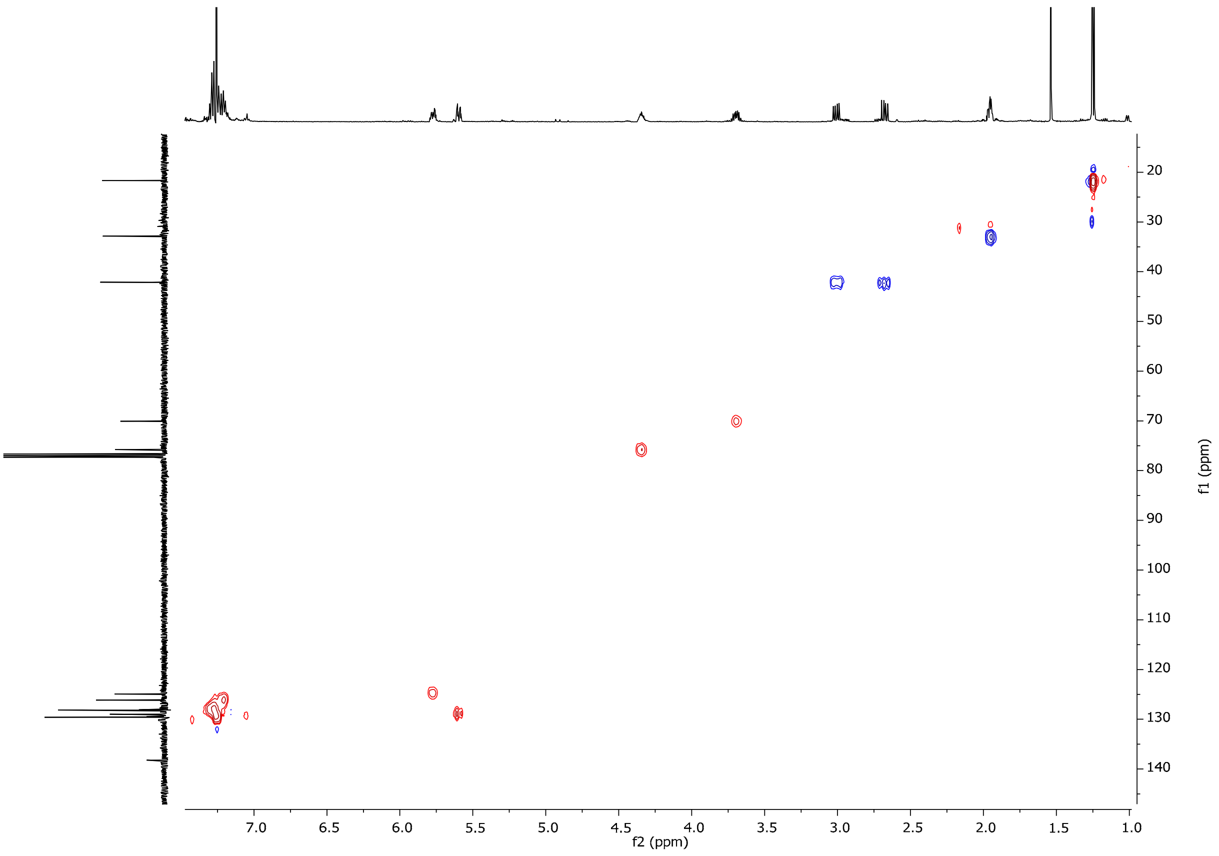

HMBC

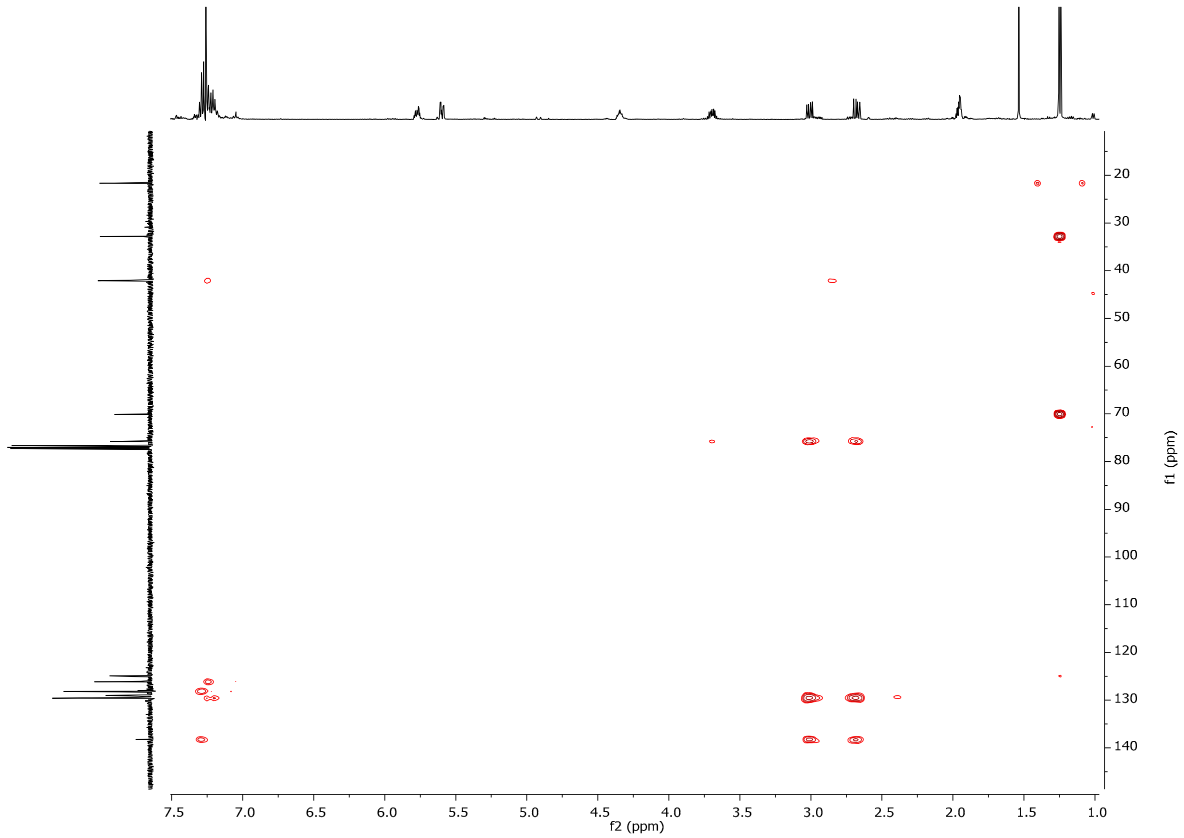

NOESY

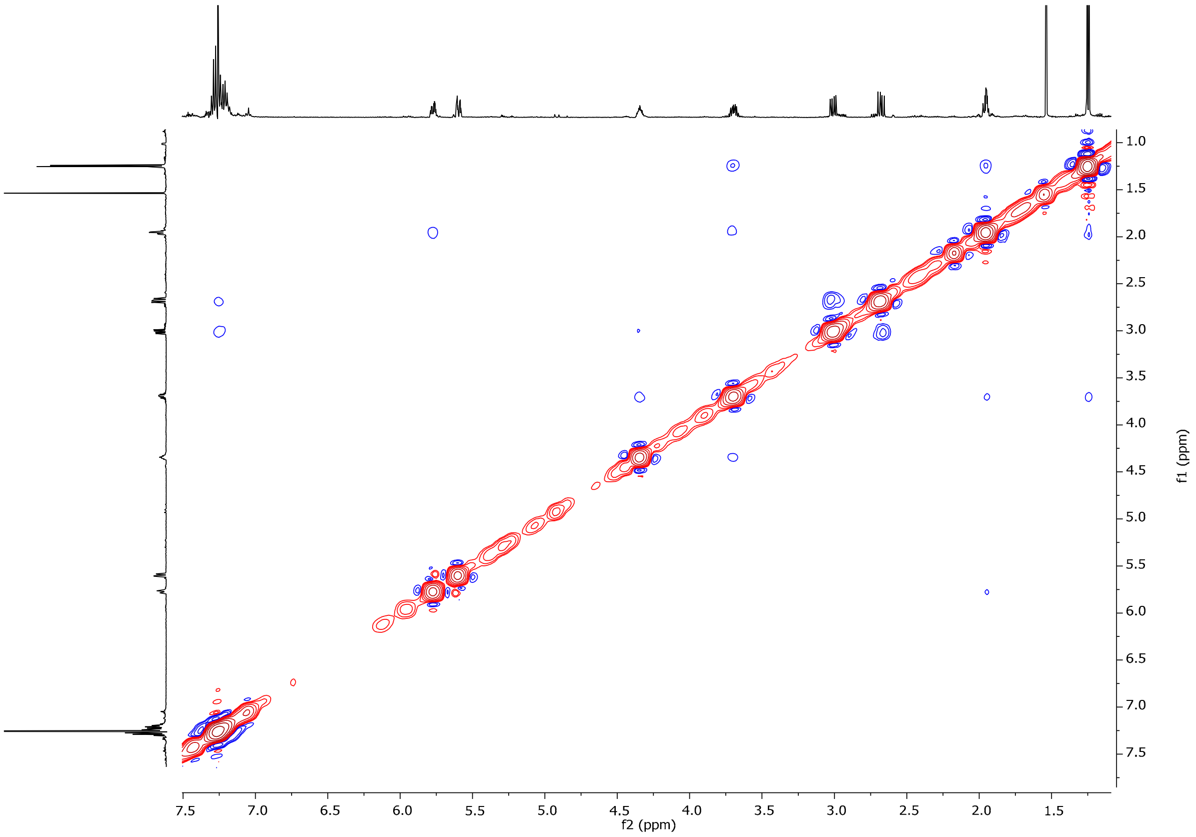

Compound 4b

^{1}H NMR (500 MHz, $CDCl_3$)

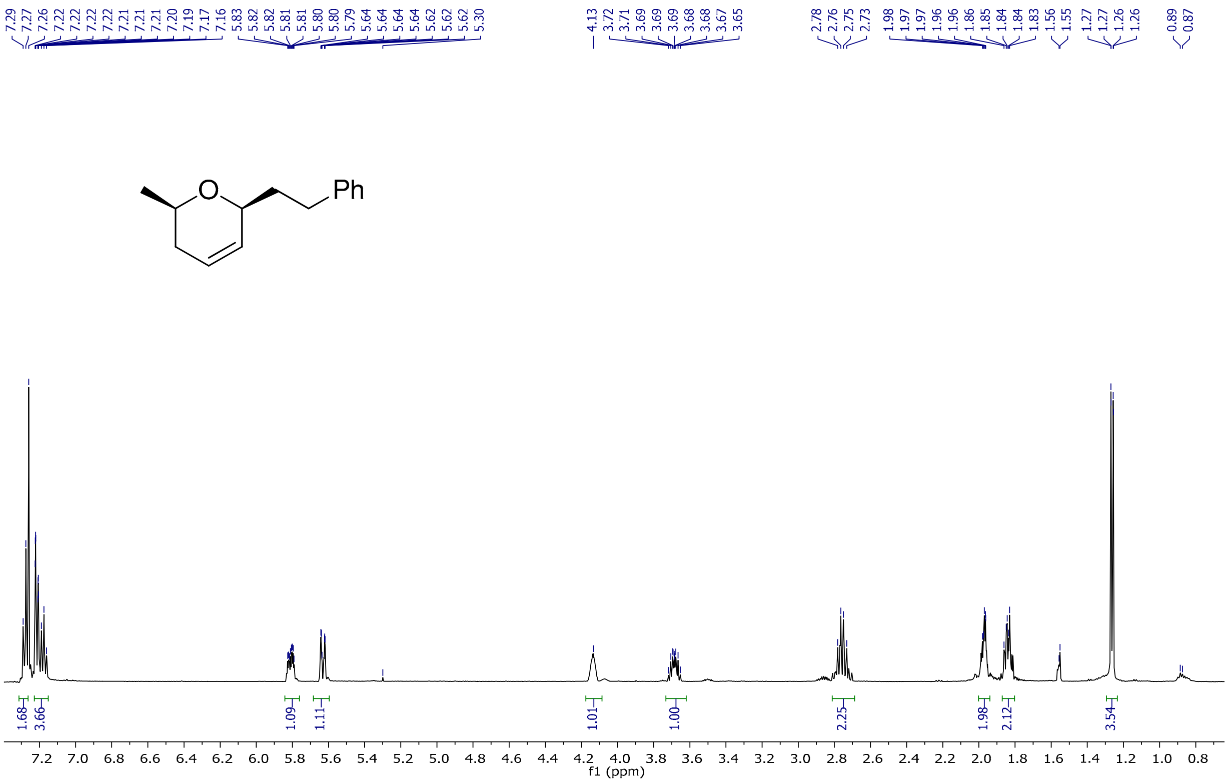

^{13}C NMR (101 MHz, $CDCl_3$)

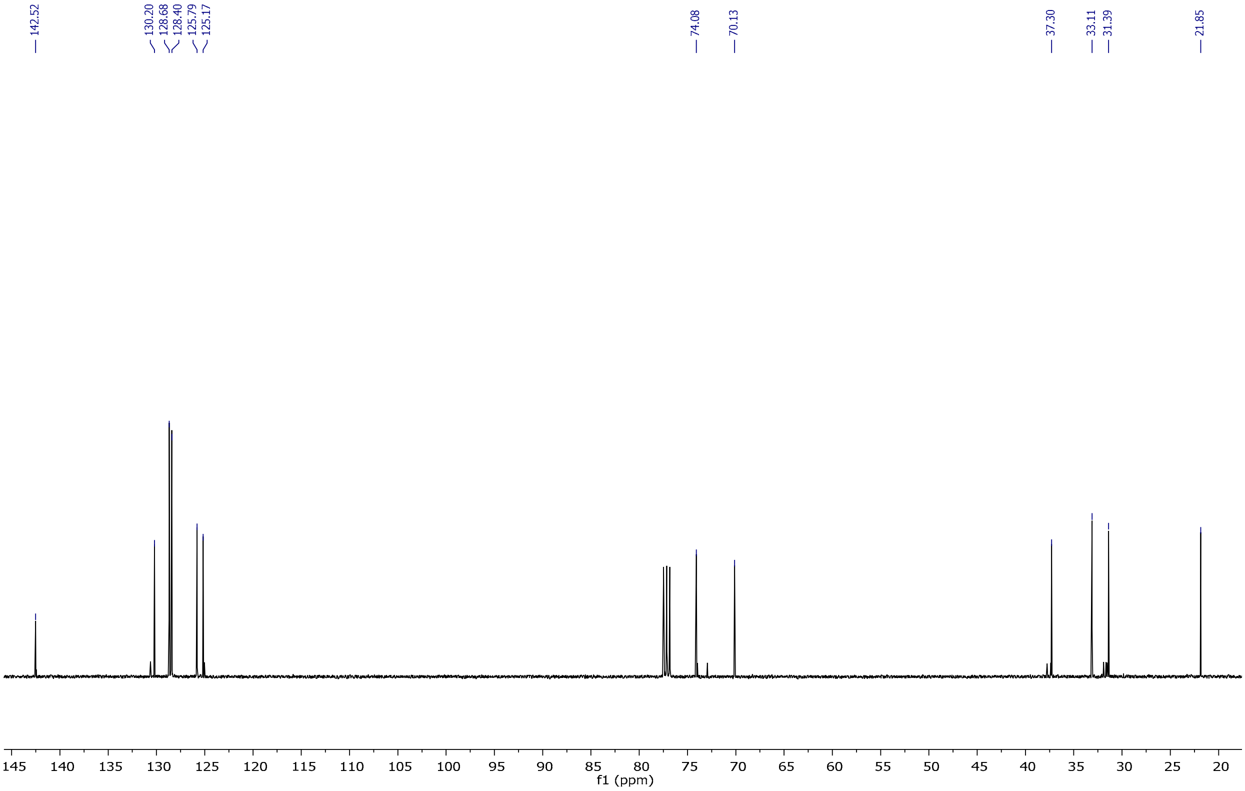

Compound 4c

^{1}H NMR (500 MHz, $CDCl_3$)

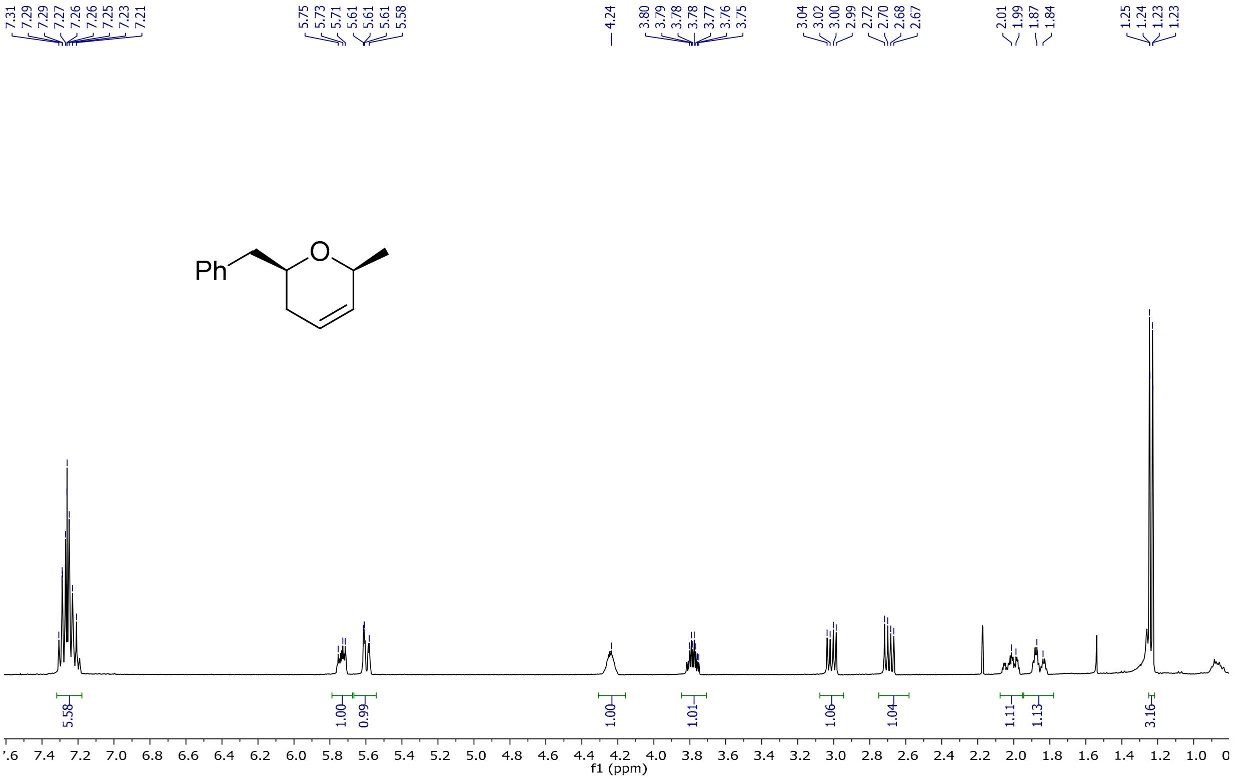

^{13}C NMR (101 MHz, $CDCl_3$)

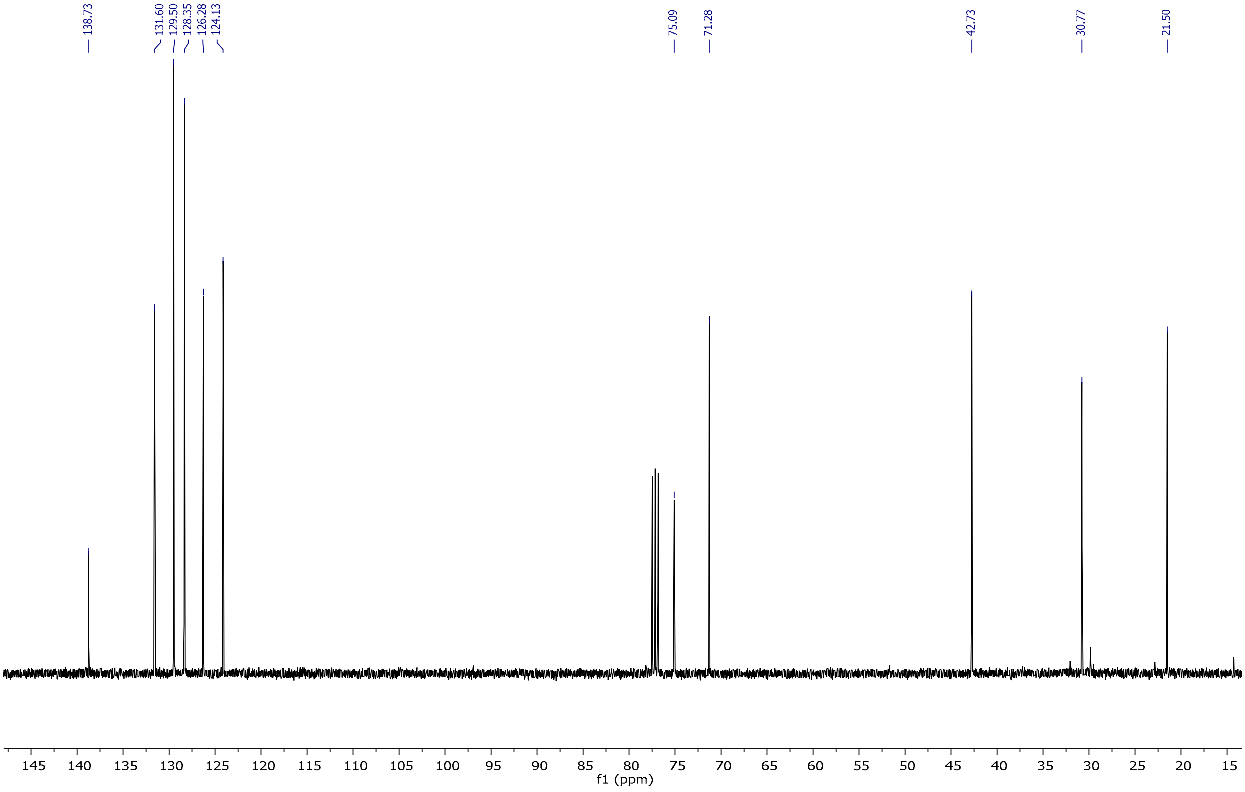

Compound 4d

^{1}H NMR (500 MHz, $CDCl_3$)

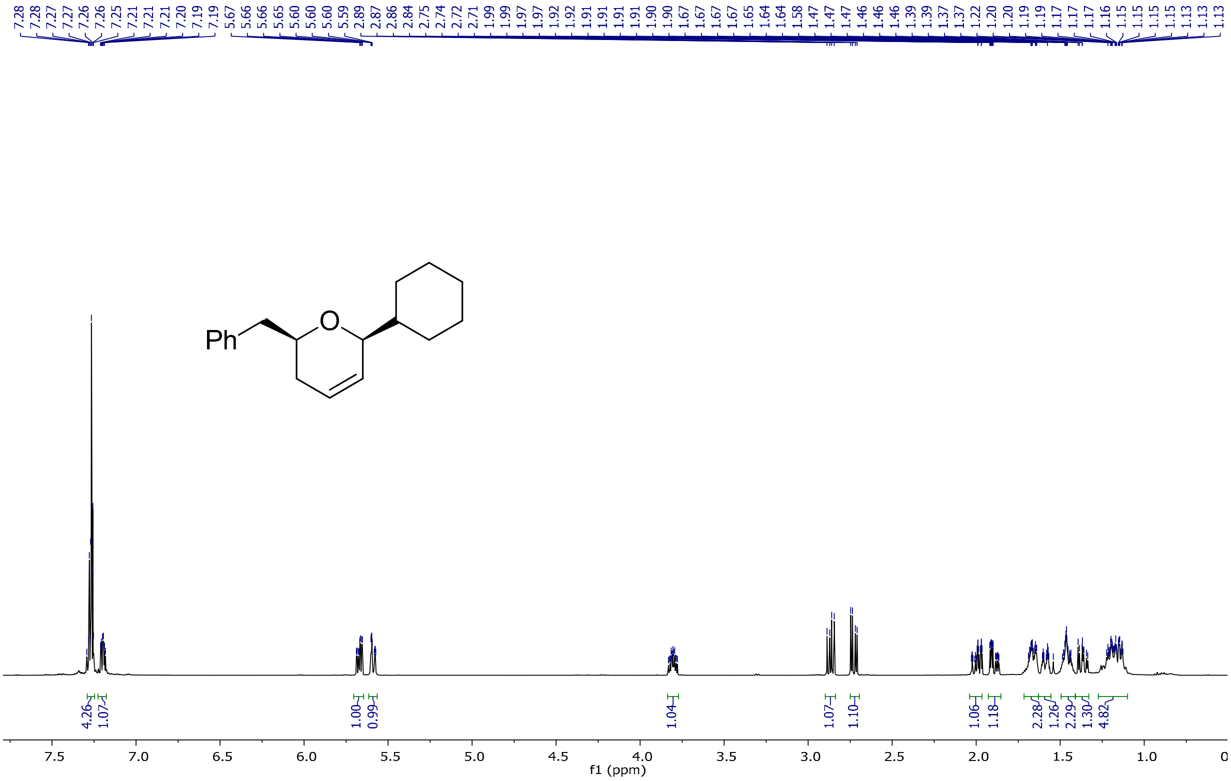

^{13}C NMR (101 MHz, $CDCl_3$)

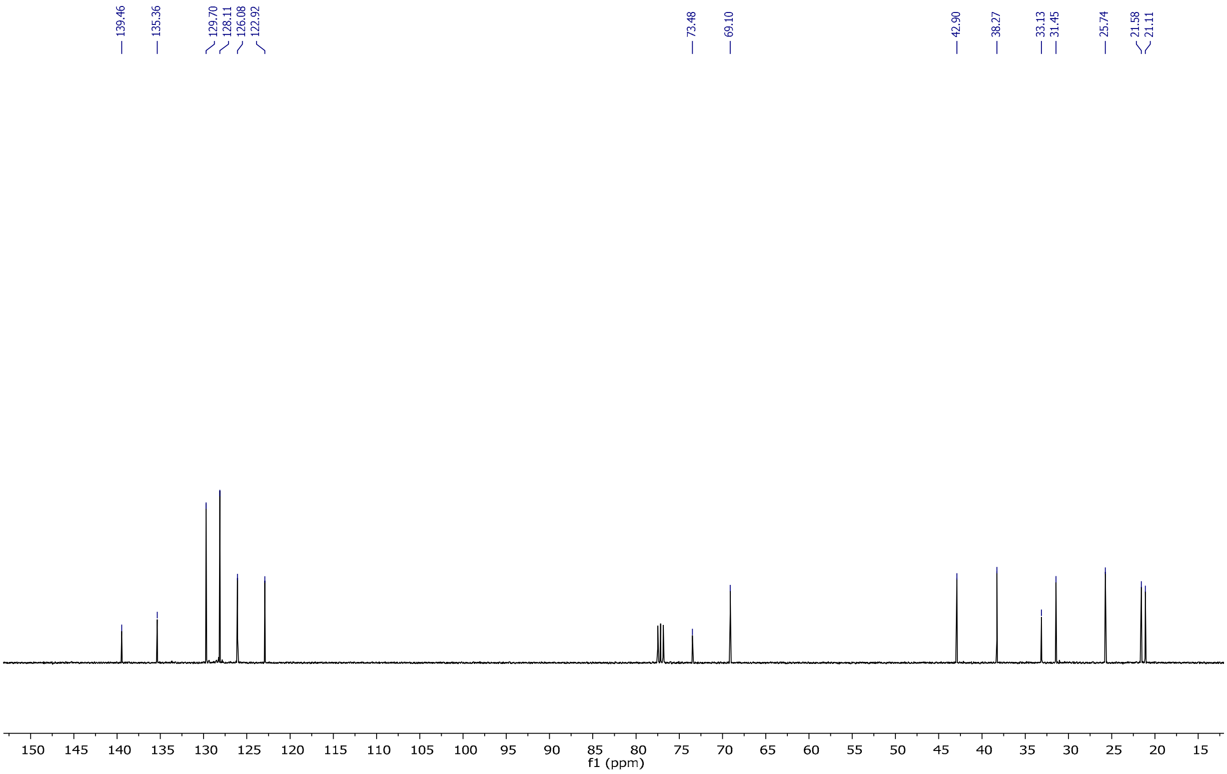

Compound 4e

^{1}H NMR (500 MHz, $CDCl_3$)

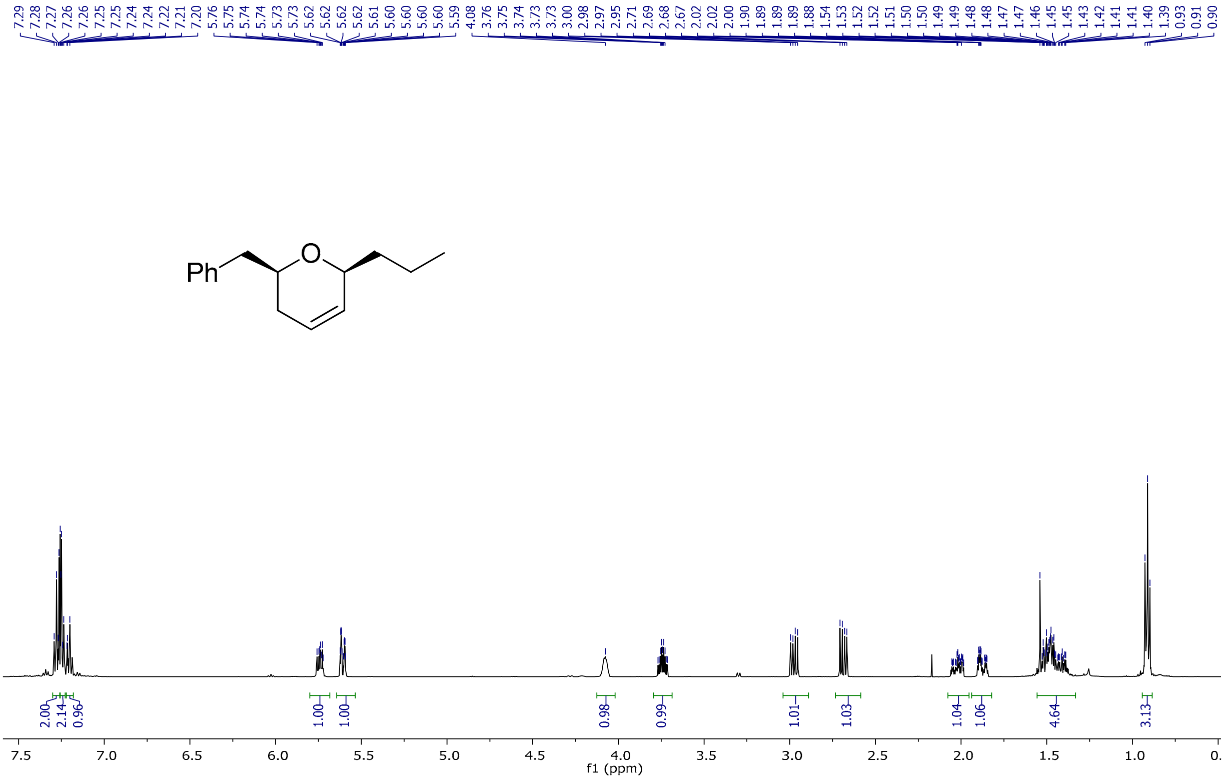

^{13}C NMR (101 MHz, $CDCl_3$)

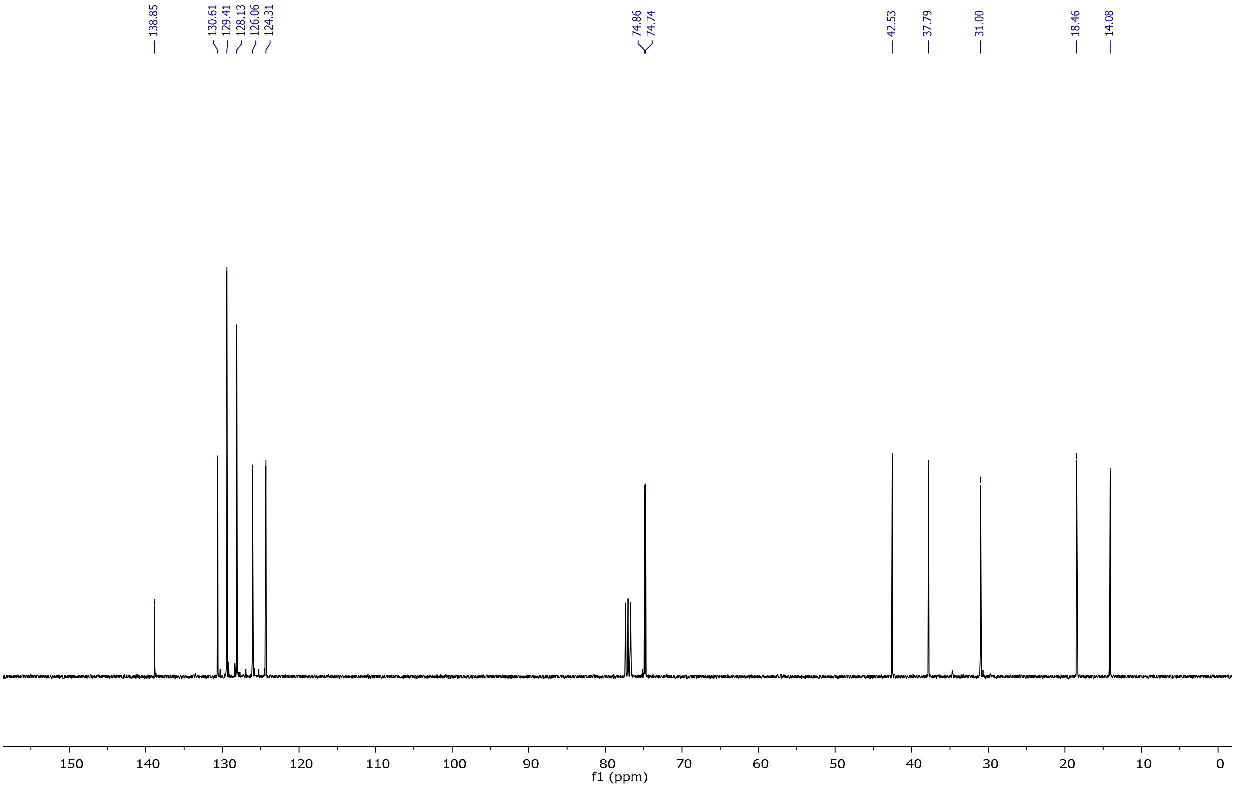

Compound 4f

^{1}H NMR (500 MHz, $CDCl_3$)

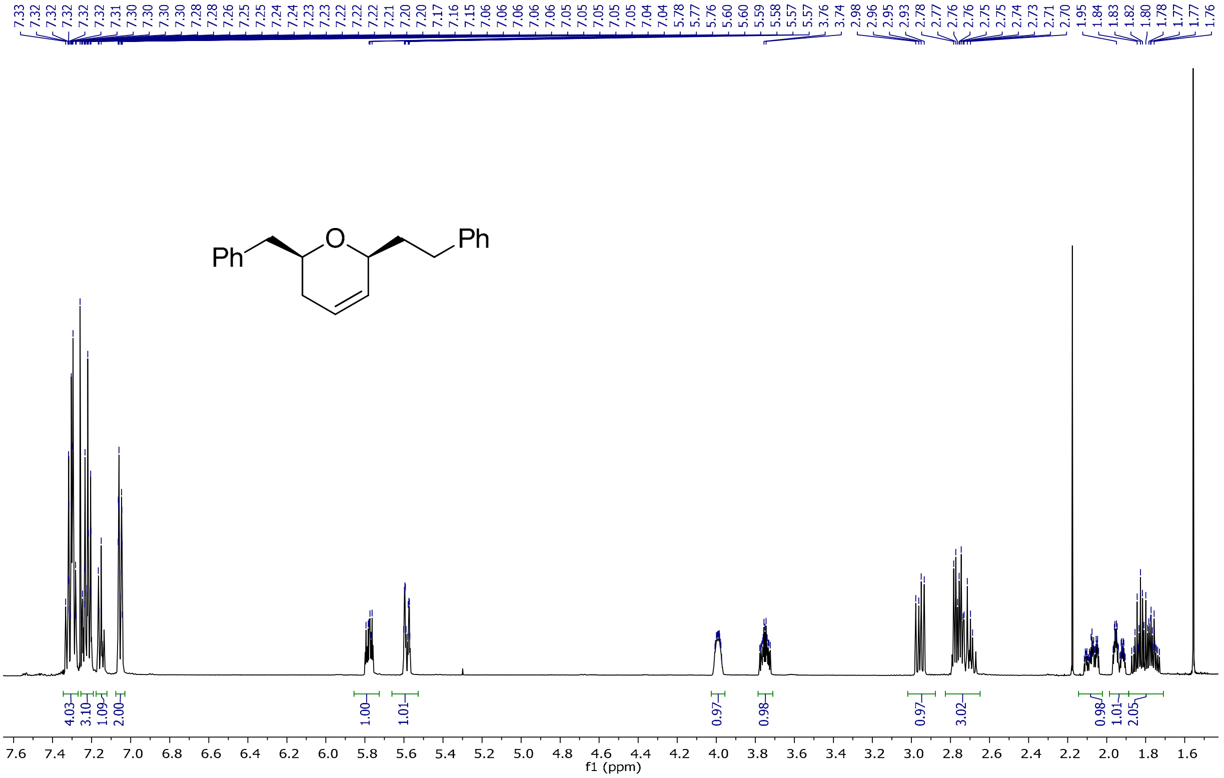

^{13}C NMR (101 MHz, $CDCl_3$)

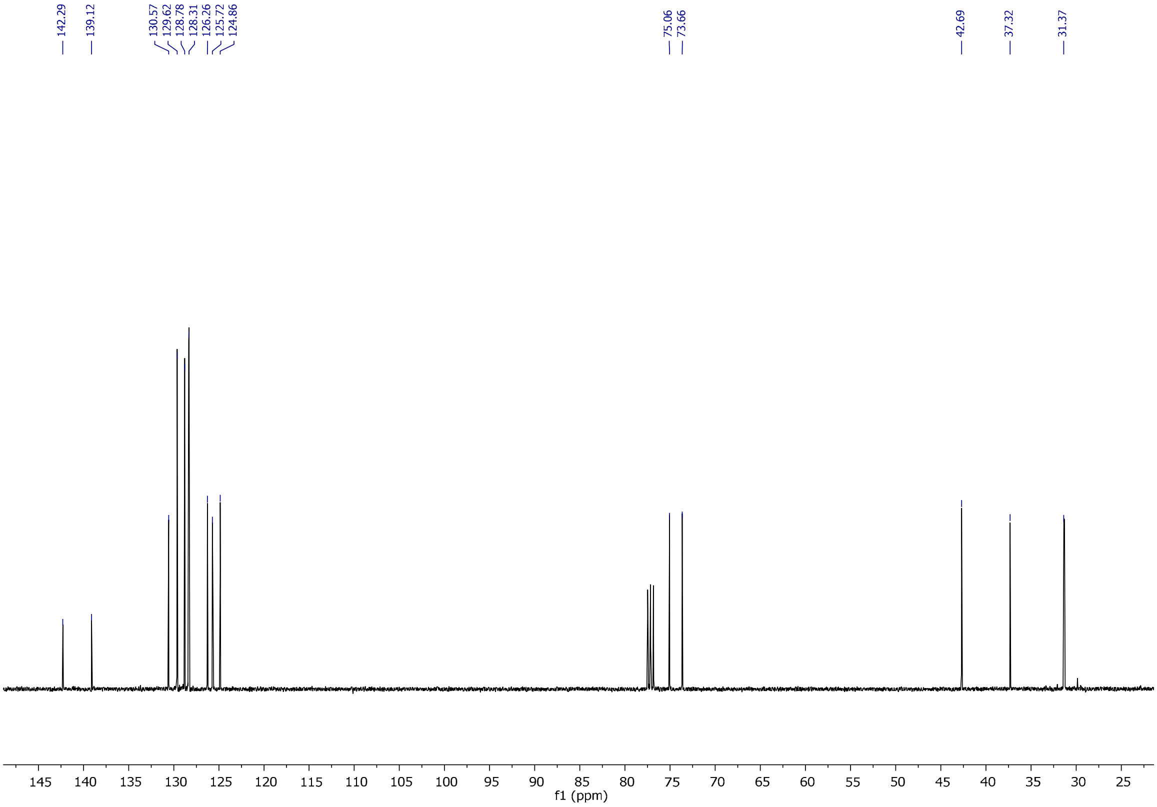

Compound 4g

^{1}H NMR (500 MHz, $CDCl_3$)

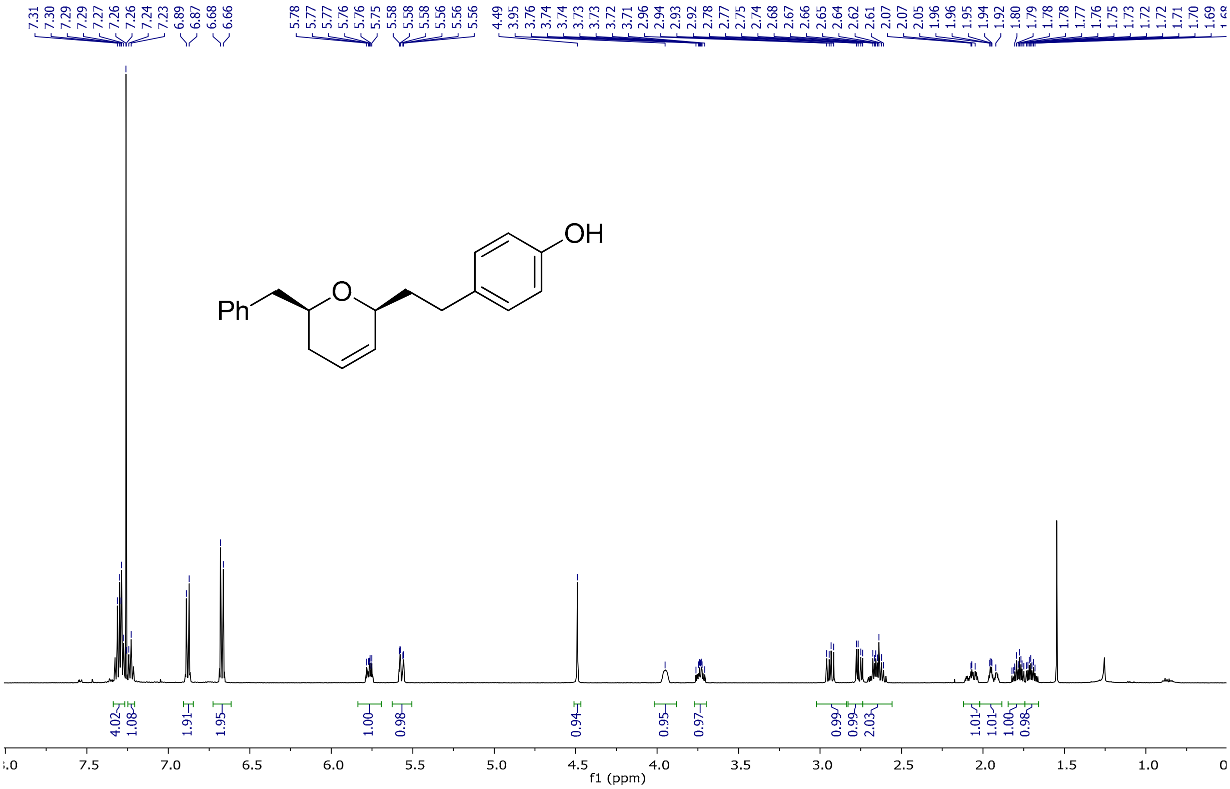

^{13}C NMR (101 MHz, $CDCl_3$)

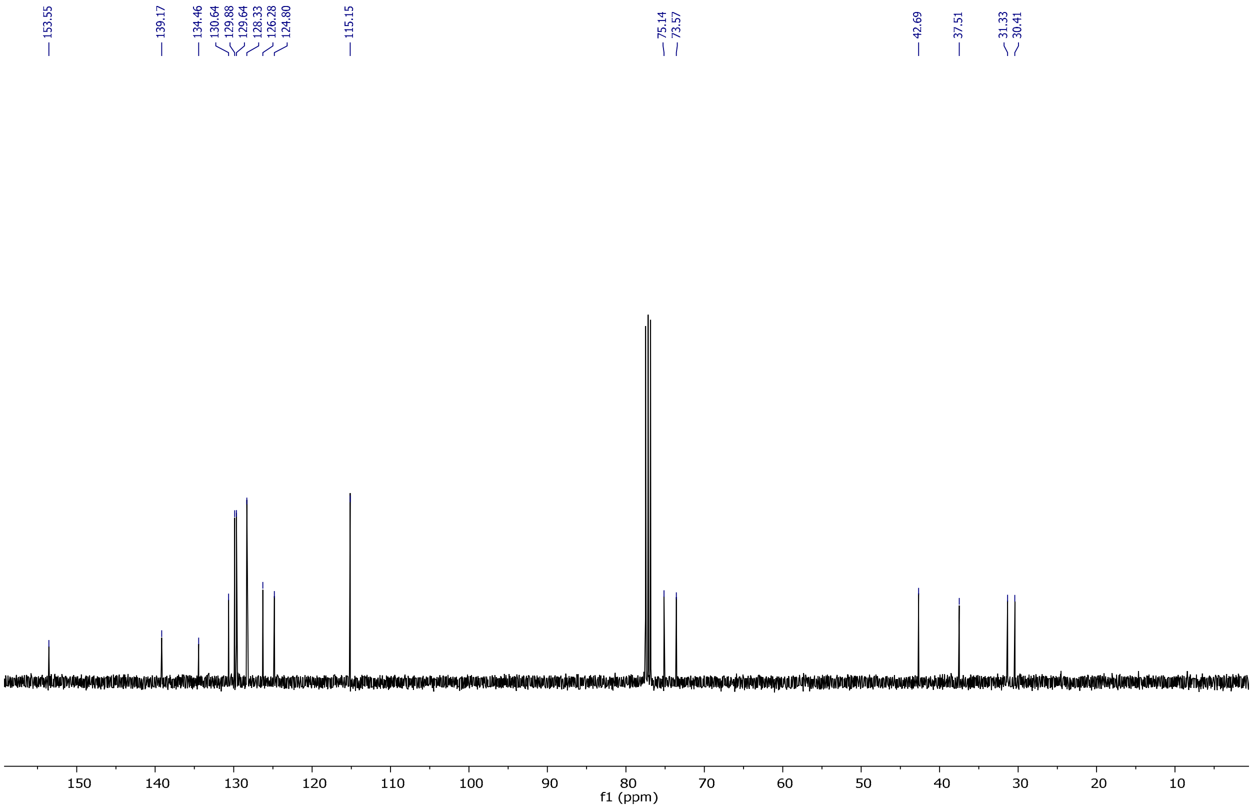

Compound 4h

^{1}H NMR (500 MHz, $CDCl_3$)

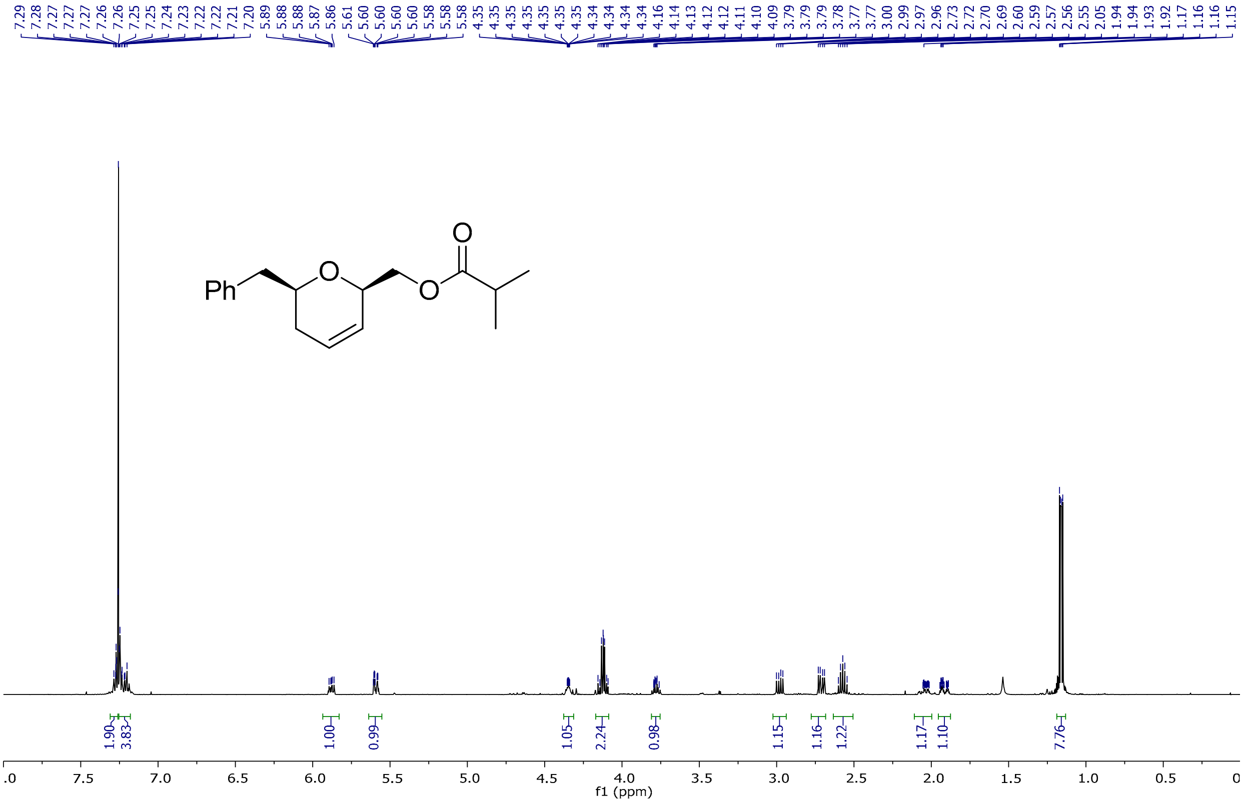

^{13}C NMR (101 MHz, $CDCl_3$)

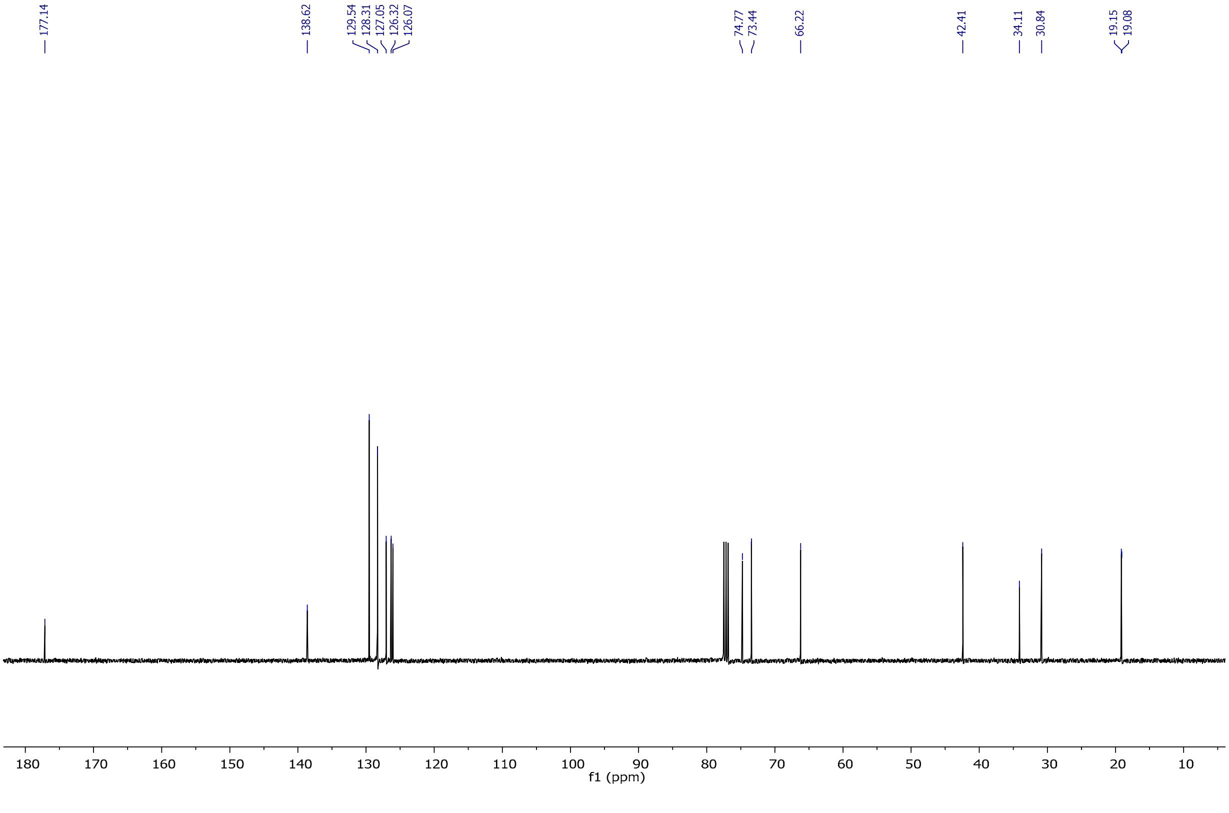

Compuesto 4i

^{1}H NMR (500 MHz, $CDCl_3$)

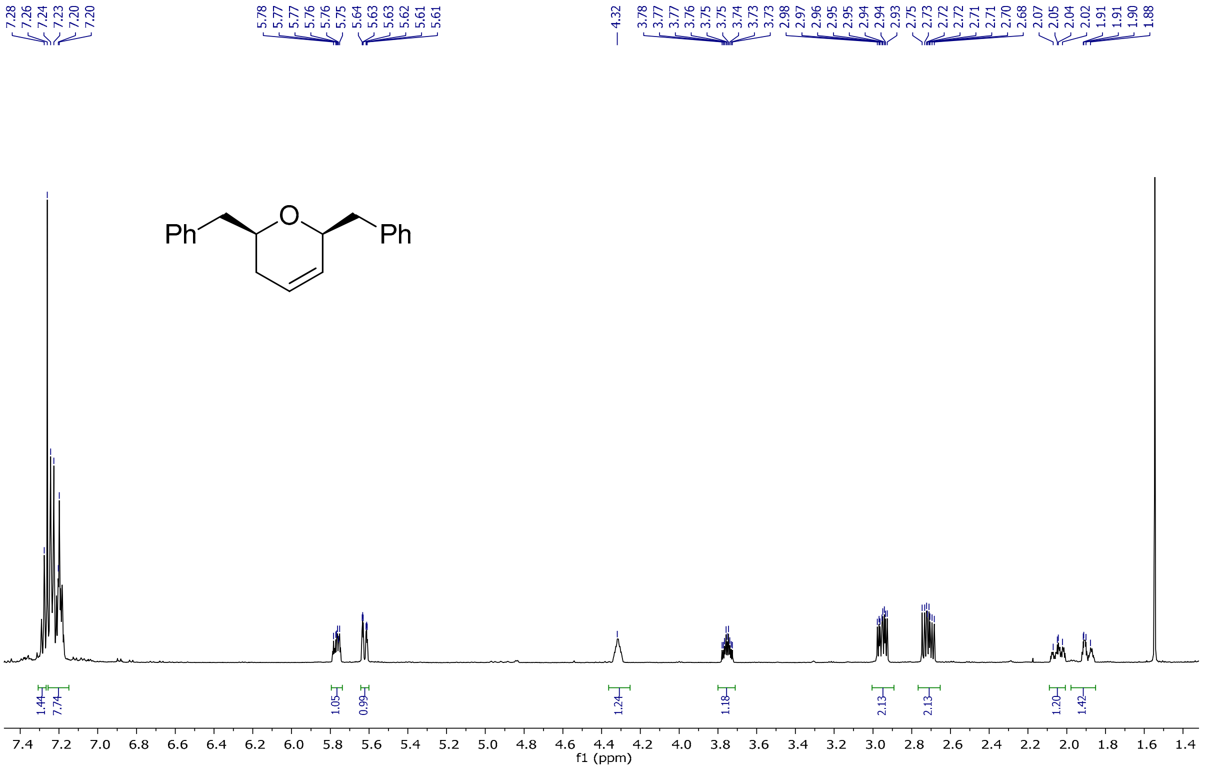

^{13}C NMR (101 MHz, $CDCl_3$)

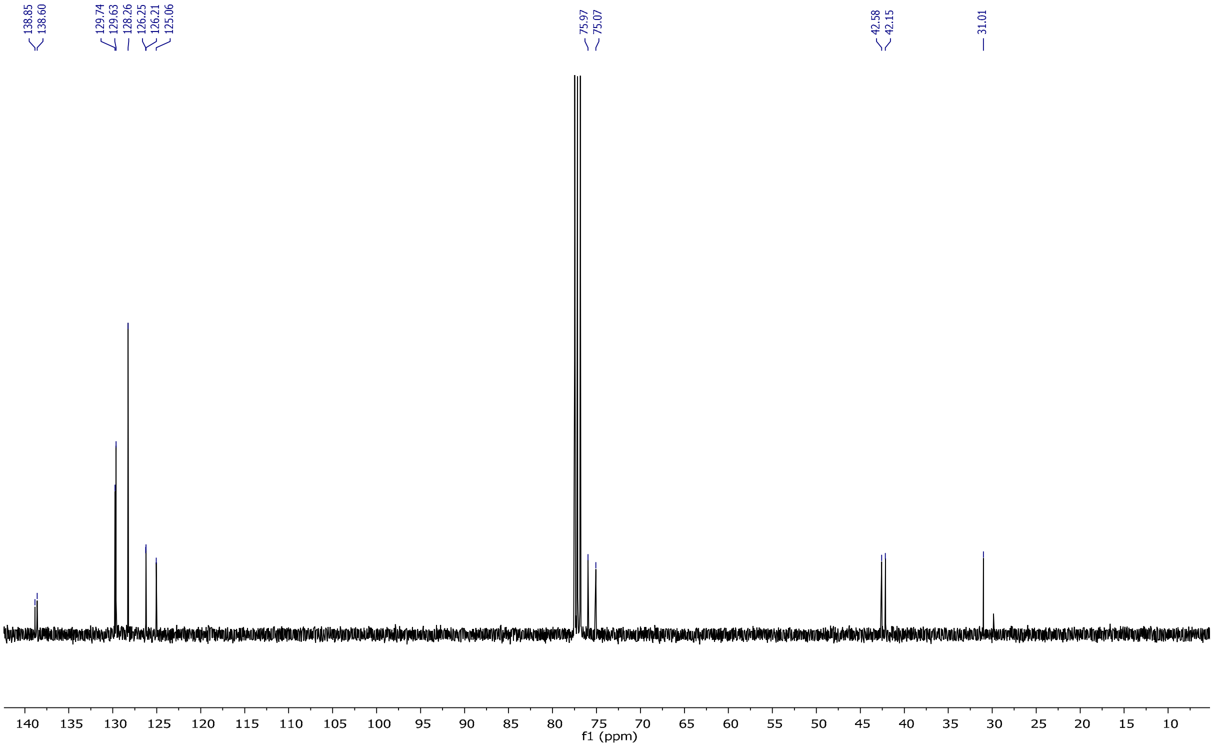

Compuesto 5

^{1}H NMR (400 MHz, $CDCl_3$)

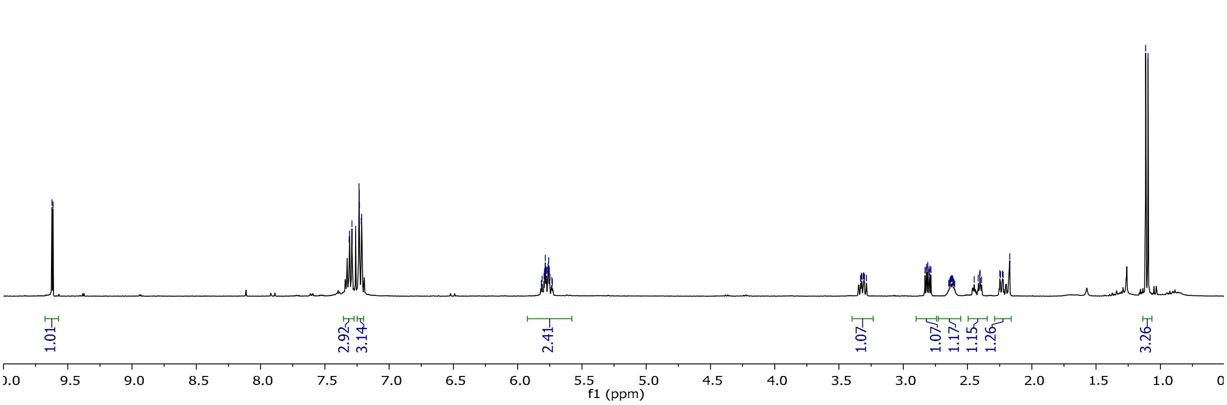

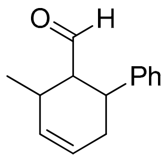

^{13}C NMR (101 MHz, $CDCl_3$)

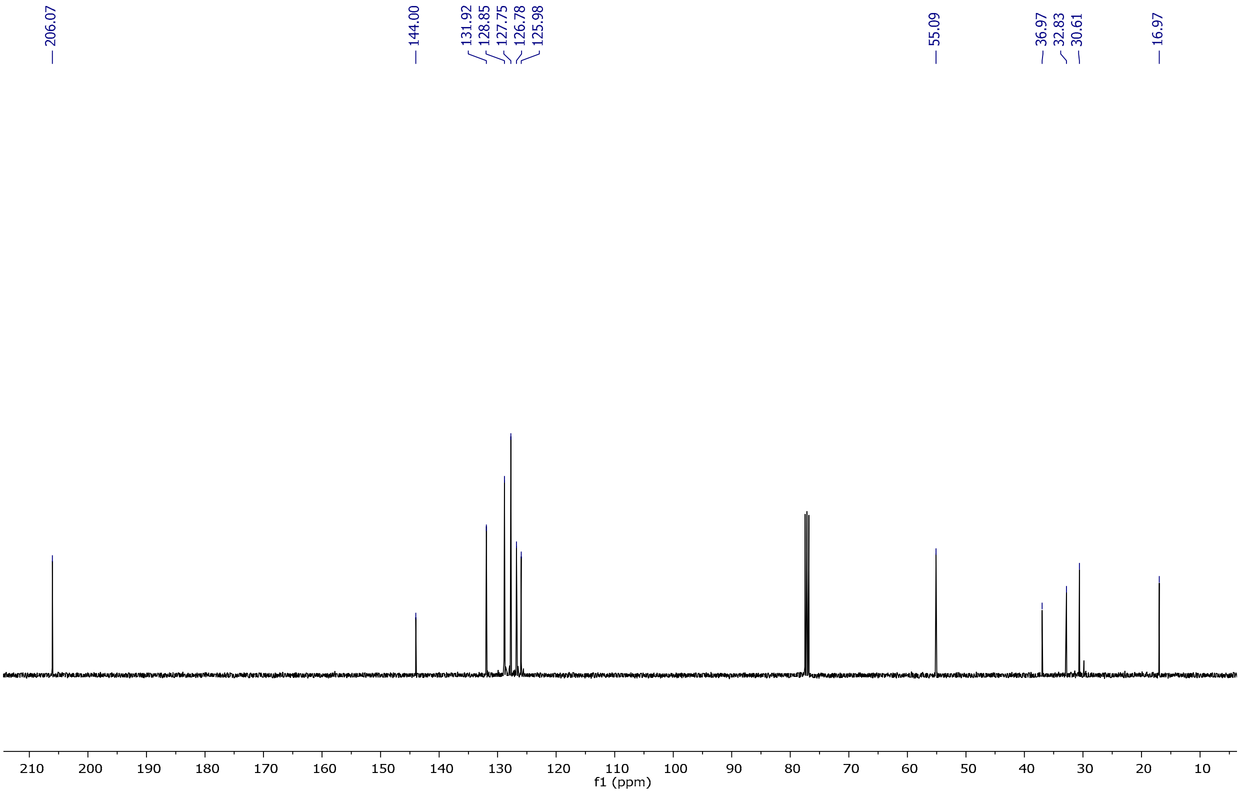

CARTESIANS COORDINATES

REACTANT (*S* isomer)

ATOM	X	Y (angstrom)	Z
C	-8.745606000	-2.158120000	-0.066294000
C	-6.398466000	-2.230557000	0.262726000
C	-7.027904000	-2.727094000	2.792025000
C	-8.064486000	-1.987599000	2.323439000
C	-9.117766000	-2.499072000	1.397595000
H	-8.587520000	-1.078299000	-0.191192000
H	-7.038564000	-3.800346000	2.558096000
H	-8.106436000	-0.914074000	2.543070000
H	-9.238272000	-3.587367000	1.481289000
H	-10.088091000	-2.019376000	1.589127000
H	-6.455589000	-1.163442000	0.498846000
O	-7.416829000	-2.813578000	-0.271730000
C	-9.688076000	-2.721627000	-1.101568000
H	-10.676368000	-2.266696000	-0.959149000
H	-9.340548000	-2.485863000	-2.114247000
H	-9.783447000	-3.809070000	-0.990089000
C	-5.079196000	-2.895447000	0.179750000
H	-4.501534000	-2.686559000	1.087470000
H	-5.195862000	-3.977909000	0.058262000
C	-4.409201000	-2.240510000	-1.028332000
C	-3.516882000	-1.173215000	-0.851471000
C	-4.726605000	-2.678951000	-2.322670000
C	-2.919825000	-0.571078000	-1.961364000
H	-3.273832000	-0.824971000	0.153932000
C	-4.125050000	-2.077641000	-3.429929000
H	-5.435393000	-3.497300000	-2.460619000
C	-3.222358000	-1.023008000	-3.250583000
H	-2.215343000	0.248907000	-1.818741000
H	-4.362414000	-2.431377000	-4.433743000
H	-2.755213000	-0.552384000	-4.116467000
Si	-5.685361000	-2.052024000	3.959131000
C	-4.298446000	-3.315118000	4.073527000
H	-3.787848000	-3.447129000	3.109054000
H	-3.547000000	-2.992700000	4.808281000
H	-4.685518000	-4.296429000	4.383413000
C	-5.096837000	-0.400991000	3.262734000
H	-4.369039000	0.060471000	3.944779000
H	-4.607910000	-0.528105000	2.285813000
H	-5.931327000	0.302865000	3.133002000
C	-6.493036000	-1.790353000	5.646350000
C	-7.370171000	-0.706695000	5.853627000
C	-6.263197000	-2.671036000	6.720492000
C	-7.994975000	-0.508474000	7.088115000
H	-7.570729000	0.000846000	5.045264000
C	-6.886027000	-2.476751000	7.958603000
H	-5.588116000	-3.520502000	6.598185000
C	-7.753499000	-1.395518000	8.143840000
H	-8.668282000	0.338789000	7.228678000
H	-6.693200000	-3.169740000	8.779323000
H	-8.239724000	-1.242654000	9.108658000

TRANSITION STATE (*S* isomer)

	angstrom		
ATOM	X	Y	Z
C	-8.630069000	-2.165738000	0.120080000
C	-6.347798000	-2.159774000	0.704990000
C	-6.769558000	-2.654600000	2.546275000
C	-8.014372000	-2.036665000	2.452083000
C	-9.084420000	-2.554131000	1.607691000
H	-8.568502000	-1.071254000	0.038922000
H	-6.769181000	-3.734758000	2.350768000
H	-8.118553000	-1.004812000	2.808774000
H	-9.169624000	-3.646505000	1.635953000
H	-10.053435000	-2.072640000	1.779484000
H	-6.363694000	-1.063390000	0.744110000
O	-7.318439000	-2.731928000	-0.041038000
C	-9.556737000	-2.759080000	-0.918701000
H	-10.570726000	-2.371656000	-0.764286000
H	-9.222297000	-2.471575000	-1.923170000
H	-9.576479000	-3.853241000	-0.840367000
C	-4.986402000	-2.772429000	0.439035000
H	-4.303016000	-2.520204000	1.255985000
H	-5.087978000	-3.864254000	0.399394000
C	-4.459254000	-2.205064000	-0.865523000
C	-3.599528000	-1.096612000	-0.856208000
C	-4.855136000	-2.757782000	-2.093006000
C	-3.128367000	-0.556004000	-2.056254000
H	-3.285147000	-0.659657000	0.093616000
C	-4.381924000	-2.220112000	-3.292306000
H	-5.531666000	-3.613797000	-2.106645000
C	-3.519353000	-1.117231000	-3.276410000
H	-2.454085000	0.301159000	-2.037217000
H	-4.686313000	-2.662686000	-4.241717000
H	-3.152190000	-0.697193000	-4.213867000
Si	-5.486865000	-1.967611000	3.822502000
C	-4.113148000	-3.230425000	4.030299000
H	-3.474310000	-3.306046000	3.140058000
H	-3.478190000	-2.940515000	4.879683000
H	-4.525840000	-4.228310000	4.236001000
C	-4.896985000	-0.289687000	3.209563000
H	-4.307456000	0.205761000	3.993917000
H	-4.258788000	-0.380572000	2.320143000
H	-5.739880000	0.369484000	2.955456000
C	-6.468590000	-1.774832000	5.419920000
C	-7.053726000	-0.542330000	5.770487000
C	-6.681897000	-2.877563000	6.271004000
C	-7.830034000	-0.415800000	6.927493000
H	-6.902037000	0.337438000	5.141144000
C	-7.455850000	-2.754905000	7.428952000
H	-6.240381000	-3.848044000	6.033506000
C	-8.032935000	-1.523105000	7.757844000
H	-8.272590000	0.548282000	7.183229000
H	-7.607829000	-3.619953000	8.076658000
H	-8.636984000	-1.426510000	8.661394000

PRODUCT (*S* isomer)

ATOM	X	Y	Z
		angstrom	
C	-8.489751000	-2.116149000	0.225531000
C	-6.222396000	-2.123768000	0.895899000
C	-6.631616000	-2.579419000	2.426039000
C	-7.943990000	-2.006545000	2.512890000
C	-9.028836000	-2.569670000	1.793836000
H	-8.474767000	-1.017942000	0.211017000
H	-6.667055000	-3.676693000	2.360469000
H	-8.042478000	-0.968677000	2.852404000
H	-9.057480000	-3.661418000	1.738825000
H	-10.007170000	-2.100335000	1.921583000
H	-6.211185000	-1.024650000	0.842879000
O	-7.222878000	-2.665012000	0.053200000
C	-9.468081000	-2.705058000	-0.766572000
H	-10.479837000	-2.336887000	-0.564154000
H	-9.173172000	-2.381264000	-1.773713000
H	-9.459119000	-3.800166000	-0.720330000
C	-4.871589000	-2.716087000	0.487290000
H	-4.126436000	-2.435535000	1.241098000
H	-4.957254000	-3.811094000	0.492726000
C	-4.451031000	-2.197852000	-0.868570000
C	-3.738637000	-0.993518000	-0.973152000
C	-4.801705000	-2.886477000	-2.039051000
C	-3.375220000	-0.488608000	-2.224976000
H	-3.459609000	-0.448790000	-0.068381000
C	-4.436288000	-2.385784000	-3.291418000
H	-5.362624000	-3.819689000	-1.966028000
C	-3.723493000	-1.184816000	-3.387464000
H	-2.816699000	0.446024000	-2.292376000
H	-4.707320000	-2.934280000	-4.194799000
H	-3.438501000	-0.794253000	-4.365400000
Si	-5.412586000	-1.945206000	3.813665000
C	-4.088964000	-3.250604000	4.059443000
H	-3.427254000	-3.342908000	3.188010000
H	-3.474347000	-2.980608000	4.930470000
H	-4.539398000	-4.234772000	4.251057000
C	-4.755469000	-0.266533000	3.286122000
H	-4.203248000	0.193983000	4.117663000
H	-4.067622000	-0.353035000	2.434057000
H	-5.566773000	0.418587000	3.000332000
C	-6.502679000	-1.785347000	5.343654000
C	-6.998136000	-0.534760000	5.762412000
C	-6.891874000	-2.930619000	6.067745000
C	-7.856762000	-0.431272000	6.862097000
H	-6.711747000	0.375455000	5.231246000
C	-7.748508000	-2.830949000	7.167574000
H	-6.524333000	-3.916905000	5.774892000
C	-8.233698000	-1.579797000	7.565322000
H	-8.228508000	0.546696000	7.171984000
H	-8.036698000	-3.728901000	7.716499000
H	-8.901295000	-1.500404000	8.424686000

CARTESIANS COORDINATES

REACTANT (*R* isomer)

ATOM	X (angstrom)	Y (angstrom)	Z (angstrom)
C	-8.749567000	-2.252107000	0.018841000
C	-6.368999000	-2.286308000	0.284477000
C	-6.944857000	-2.832933000	2.795268000
C	-8.059738000	-2.156117000	2.421571000
C	-9.085335000	-2.684077000	1.473715000
H	-9.375729000	-2.804782000	-0.687185000
H	-6.866267000	-3.883021000	2.482695000
H	-8.191258000	-1.119253000	2.752258000
H	-9.128681000	-3.779692000	1.504863000
H	-10.085613000	-2.284687000	1.691742000
H	-6.413819000	-1.234705000	0.577371000
O	-7.399385000	-2.854612000	-0.237841000
C	-8.788487000	-0.764645000	-0.259893000
H	-9.838894000	-0.448519000	-0.232352000
H	-8.247269000	-0.166551000	0.482977000
H	-8.393856000	-0.549463000	-1.260490000
C	-5.052779000	-2.944838000	0.128770000
H	-4.458460000	-2.807098000	1.039846000
H	-5.176000000	-4.015141000	-0.070742000
C	-4.401607000	-2.202169000	-1.037984000
C	-3.437081000	-1.212041000	-0.801807000
C	-4.807521000	-2.482413000	-2.351745000
C	-2.858775000	-0.530608000	-1.875125000
H	-3.121771000	-0.988108000	0.218573000
C	-4.225644000	-1.801272000	-3.422743000
H	-5.571612000	-3.240029000	-2.534553000
C	-3.251796000	-0.824493000	-3.185568000
H	-2.097958000	0.227706000	-1.687699000
H	-4.533995000	-2.031894000	-4.442929000
H	-2.799479000	-0.291211000	-4.022499000
Si	-5.626956000	-2.088296000	3.951275000
C	-4.238418000	-3.338835000	4.149400000
H	-3.743849000	-3.544432000	3.188789000
H	-3.478623000	-2.961209000	4.848010000
H	-4.619773000	-4.293883000	4.538224000
C	-5.032341000	-0.477028000	3.169879000
H	-4.366042000	0.062805000	3.857282000
H	-4.471077000	-0.666704000	2.243734000
H	-5.873976000	0.186406000	2.923155000
C	-6.472134000	-1.733036000	5.601006000
C	-7.039791000	-0.471965000	5.869399000
C	-6.599256000	-2.738626000	6.579859000
C	-7.712614000	-0.223577000	7.070505000
H	-6.955133000	0.335343000	5.138608000
C	-7.269487000	-2.494546000	7.782660000
H	-6.170246000	-3.728446000	6.408217000
C	-7.829312000	-1.235887000	8.028996000
H	-8.143032000	0.761259000	7.259963000
H	-7.354950000	-3.286312000	8.528881000
H	-8.352847000	-1.043877000	8.966933000

PRODUCT (*R* isomer)

ATOM	X	angstrom<br>Y	Z
C	-8.475572000	-2.262649000	0.259413000
C	-6.180225000	-2.149673000	0.904088000
C	-6.561397000	-2.621031000	2.444819000
C	-7.900975000	-2.123253000	2.552248000
C	-8.957230000	-2.740104000	1.827046000
H	-9.112486000	-2.899865000	-0.364964000
H	-6.538238000	-3.718059000	2.368673000
H	-8.054625000	-1.102935000	2.920247000
H	-8.919849000	-3.831794000	1.778116000
H	-9.962999000	-2.339103000	1.974402000
H	-6.186348000	-1.052193000	0.859156000
O	-7.168658000	-2.725774000	0.076218000
C	-8.726203000	-0.789642000	-0.005224000
H	-9.790286000	-0.570021000	0.142568000
H	-8.147550000	-0.120921000	0.640878000
H	-8.471996000	-0.577329000	-1.051767000
C	-4.817145000	-2.708268000	0.488789000
H	-4.075937000	-2.417148000	1.242919000
H	-4.878053000	-3.804850000	0.483751000
C	-4.415287000	-2.167095000	-0.863766000
C	-3.751515000	-0.934752000	-0.961893000
C	-4.737746000	-2.863123000	-2.037941000
C	-3.408296000	-0.409283000	-2.210773000
H	-3.493502000	-0.384083000	-0.054558000
C	-4.392204000	-2.341759000	-3.287518000
H	-5.260450000	-3.818688000	-1.970111000
C	-3.728416000	-1.112643000	-3.377057000
H	-2.886953000	0.546946000	-2.272927000
H	-4.641084000	-2.896000000	-4.193750000
H	-3.459043000	-0.705823000	-4.352788000
Si	-5.367095000	-1.917779000	3.817739000
C	-4.036309000	-3.202200000	4.128628000
H	-3.424385000	-3.394749000	3.236917000
H	-3.373256000	-2.852931000	4.933448000
H	-4.491616000	-4.150532000	4.447984000
C	-4.708491000	-0.249461000	3.261738000
H	-4.107162000	0.199513000	4.065323000
H	-4.061559000	-0.338309000	2.378932000
H	-5.526871000	0.445922000	3.025461000
C	-6.501225000	-1.714092000	5.312967000
C	-6.560542000	-0.515262000	6.046646000
C	-7.342384000	-2.776841000	5.705891000
C	-7.430784000	-0.380052000	7.134618000
H	-5.929199000	0.330878000	5.768847000
C	-8.214274000	-2.645198000	6.790325000
H	-7.318665000	-3.726439000	5.164283000
C	-8.258759000	-1.443849000	7.506833000
H	-7.465686000	0.559166000	7.688901000
H	-8.856370000	-3.479049000	7.078138000
H	-8.938712000	-1.337411000	8.353395000

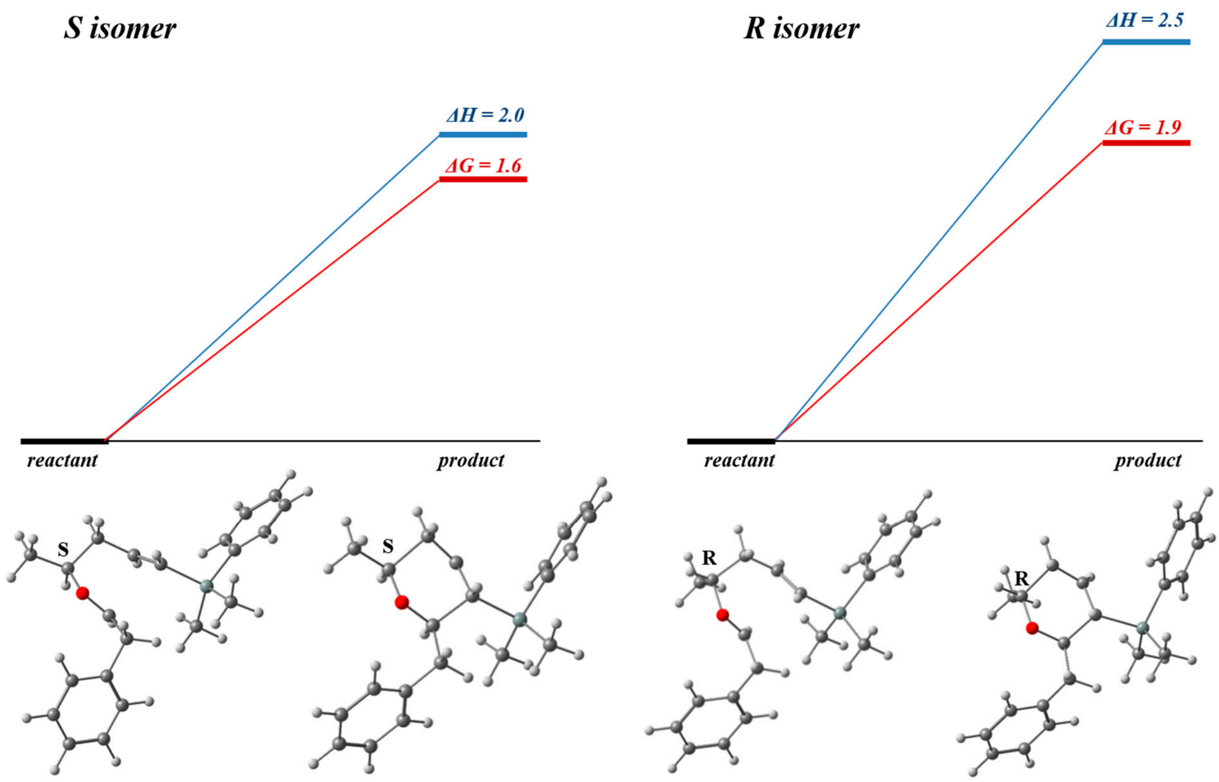

Figure S1. Relative energies for Reactants and products for *S* isomer and *R* isomer respectively computed at BP86-D3 triple-z theoretical level

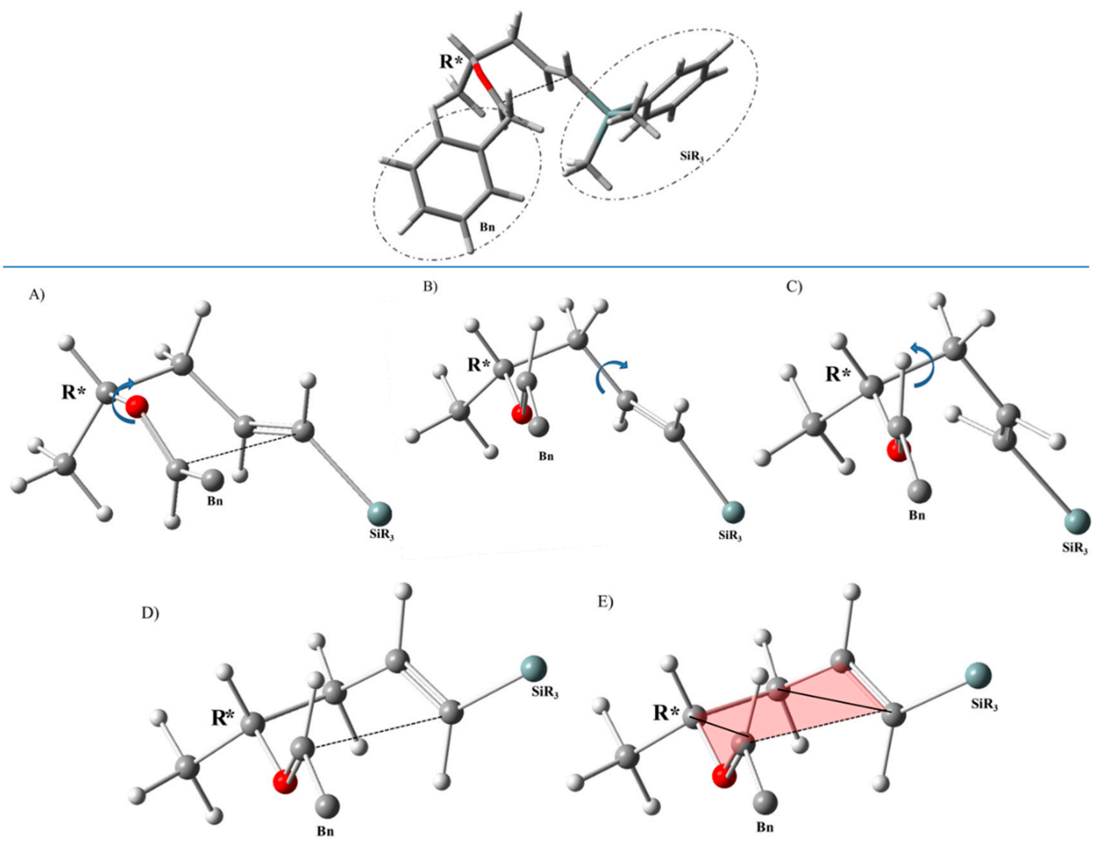

Scheme S1. Sequence of consecutive rotations for sigma bonds that place bulky groups in equatorial arrangement for *R* isomer.
